# Human and Non-Human Primate Genomes Share Hotspots of Positive Selection

**DOI:** 10.1371/journal.pgen.1000840

**Published:** 2010-02-05

**Authors:** David Enard, Frantz Depaulis, Hugues Roest Crollius

**Affiliations:** 1 DYOGEN Lab, CNRS UMR8541, Ecole Normale Supérieure, Paris, France; 2 Laboratoire Ecologie et Evolution, CNRS UMR7625, Ecole Normale Supérieure, UPMC Paris Universitas, Paris, France; University of Chicago, United States of America

## Abstract

Among primates, genome-wide analysis of recent positive selection is currently limited to the human species because it requires extensive sampling of genotypic data from many individuals. The extent to which genes positively selected in human also present adaptive changes in other primates therefore remains unknown. This question is important because a gene that has been positively selected independently in the human and in other primate lineages may be less likely to be involved in human specific phenotypic changes such as dietary habits or cognitive abilities. To answer this question, we analysed heterozygous Single Nucleotide Polymorphisms (SNPs) in the genomes of single human, chimpanzee, orangutan, and macaque individuals using a new method aiming to identify selective sweeps genome-wide. We found an unexpectedly high number of orthologous genes exhibiting signatures of a selective sweep simultaneously in several primate species, suggesting the presence of hotspots of positive selection. A similar significant excess is evident when comparing genes positively selected during recent human evolution with genes subjected to positive selection in their coding sequence in other primate lineages and identified using a different test. These findings are further supported by comparing several published human genome scans for positive selection with our findings in non-human primate genomes. We thus provide extensive evidence that the co-occurrence of positive selection in humans and in other primates at the same genetic loci can be measured with only four species, an indication that it may be a widespread phenomenon. The identification of positive selection in humans alongside other primates is a powerful tool to outline those genes that were selected uniquely during recent human evolution.

## Author Summary

An advantageous mutation spreads from generation to generation in a population until individuals that carry it, because of their higher reproductive success, completely replace those that do not. This process, commonly known as positive Darwinian selection, requires the selected mutation to induce a new non-neutral heritable phenotypic trait, and this has been shown to occur unexpectedly frequently during recent human evolution. Although the exact advantageous mutation is difficult to identify, it leaves a wider footprint on neighbouring linked neutral variation called a selective sweep. We have developed an empirical method that uses whole-genome shotgun sequences of single individuals to detect selective sweeps. By doing so, we were able to extend to chimpanzee, orangutan, and macaque individuals analyses of recent positive selection that until now were only available for human. Comparisons of genes candidates for positive selection between human and non-human primates then revealed an unexpectedly high number of cases where a selective sweep at a gene in humans is mirrored by independent positive selection at the same gene in multiple other primates. This result has future implications for understanding the nature of biological changes that underlie selective sweeps in humans.

## Introduction

The respective contribution of neutral and advantageous mutations to genetic differences between species has been a pivotal question in molecular evolution for more than half a century [1]. Until recently, results were based on typically small genetic samples leading to controversial conclusions. Only during the past decade did large genetic variation datasets make it possible to estimate reliable distributions of fitness effects [2] for a series of species such as drosophila [3] and human [3],[4]. Although estimating this distribution is not trivial under complex demographic histories and although differences remain between studies on details, different approaches now converge to conclude that a substantial proportion of non-deleterious mutations are indeed weakly to strongly advantageous [4]–[9]. In drosophila, it was found that between approximately 25% and 50% of amino-acid substitutions [6],[7] and 20% of intergenic substitutions [8] may be adaptive. In human where effective population size is smaller, estimated proportions vary from 10% to 20% [4],[10].

Such substantial proportions agree well with several scans for selective sweeps in the human genome concluding that selective sweeps are common and affect human genetic diversity [9], [11]–[19]. This may however seem contradictory with results from methods based on non-synonymous versus synonymous divergence analyses in coding sequences, such as PAML site and branch-site likelihood ratio tests for positive selection. Indeed, the PAML branch-site test 2 infers positive selection in the human lineage following divergence with chimpanzee for far fewer genes than scans for selective sweeps [20]–[24], despite the fact that such scans examine a comparatively much narrower evolutionary period. However, site and branch-site tests for positive selection are generally conservative and coding sequences represent only a small part of mammalian genomes, thus explaining much of the differences between the two approaches. Despite their conservativeness, site tests for positive selection were recently able to show that hundreds of coding sequences experienced multiple rounds of positive selection during mammalian evolution [22]. Scans for selective sweeps nevertheless capture many more adaptive events and, together with an increasing number of striking cases of parallel/convergent adaptive evolution [25]–[32], suggest that the current view of the quantitative importance of positive selection acting at the same locus independently in distinct species is still underestimated.

This question is of particular interest in the context of recent human evolution. Here and in the rest of this manuscript “recent” means detectable at the genetic intraspecific variation level, as opposed to positive selection detectable at the divergence level. It is currently unknown (i) which proportion of genes were positively selected recently in human but also experienced positive selection in other primate lineages, either recently or within a more extensive evolutionary time and (ii) which genes were in contrast positively selected only in modern human. The distinction is important to unravel the plausible nature and “uniqueness” of adaptive changes that underlie selective sweeps. For example if a selective sweep is found for a gene in human, it is often tempting to first examine if this gene governs a specifically human phenotype, and if so to interpret the sweep in terms of a strictly human-specific adaptation. But knowing that orthologs of this gene are also associated with positive selection in other primates, although not excluding the possibility of a human-specific adaptation (same gene, human-specific nature of phenotypic change), might more accurately redirect interpretations on the nature of adaptation towards scenarios that are not restricted to human-specific phenotypes (same gene, similar nature of phenotypic change in human and other primates).

Here, we estimated the quantitative importance of positive selection acting on the same genes independently in human evolution and three other primate lineages, either recently or across more extensive evolutionary times (chimpanzee, orangutan and macaque). Because genome wide genotyping datasets such as those provided by the HapMap [33] and Perlegen [34] projects for human populations are not available for non-human primates, we have developed an empirical method that detects candidate selective sweeps using complete genomes of single individuals from natural populations (see Methods). We exploited the fact that alleles linked to a positively selected mutation also increase in frequency through genetic hitchhiking [35], which results in a loss of variants unlinked to the selected haplotypes and thus in a reduction of the surrounding genetic diversity that defines a selective sweep. Our results show that positive selection affecting the same genes independently in human and other primates is (i) a common phenomenon and (ii) is not restricted to specific functions such as defence against pathogens or reproduction.

## Results

### Human selective sweeps from the point of view of two individuals

**Figure 1:**
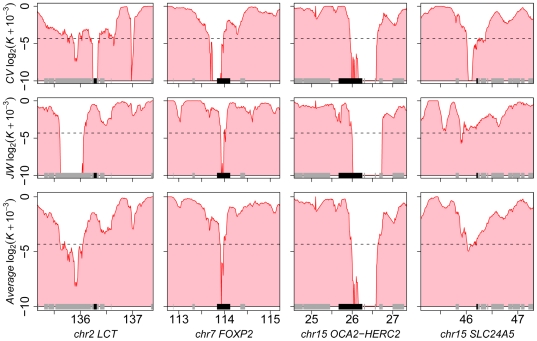
Known selective sweeps identified in individual human genomes. From left to right, each graph presents the variation of *K* at four known examples of selective sweeps in the European human population, namely the lactase gene *LCT*, *FOXP2*, *OCA2-HERC2* and *SLC24A5*. The upper, middle and lower panels show genome scans (200 kb windows in steps of 10 kb) for CV, JW and the average between both individuals, respectively. The X axis indicates chromosome coordinates in megabases, the Y axis indicates the log_2_ of *K* (+0.001 to avoid null values). Dotted horizontal lines delineate the *K* = 0.05 threshold. Genes are highlighted in grey above the X axis, with the gene known to be affected by positive selection highlighted in black.

Our method is inspired by the HKA test [19],[36] and contrasts the heterozygosity measured in a local genomic window with the value measured within its surrounding genomic context, while using inter species divergence to control for variable neutral mutation rate (including selective constraint). The corrected level of heterozygosity is indicated by the value of a statistic *K* computed for each window (0≤*K*≤1). The method thus exploits the localized nature of the hitchhiking effect of an adaptive mutation, and controls for natural and experimental factors known to influence the observed genetic diversity in primate genome sequences obtained by shotgun sequencing (see Methods; Text S1 and Table S1). We first validated the method using extensive forward population simulations [37]–[40] (Text S2, Figures S1, S2, S3), and applied the method independently to two individual human genome sequences [41],[42] (respectively J. Craig Venter (CV) and James Watson (JW)). First, we find extensive overlap (24%; co-occurrence test P<10^−5^; see Methods) between the 2,244 and 2,193 genes identified in the respective genomes of CV and JW with *K*≤0.05. This is expected if the method correctly identifies genes with reduced heterozygosity due either to selective sweeps or shared demographic history. Second, genes detected with *K*≤0.05 when averaging both individuals include well known examples of recent positive selection in Europeans, such as the *FOXP2* [43], *OCA2-HERC2* [12],[44] and *SLC24A5* [14],[45] loci (Figure 1, Table S2). Interestingly, we identified a sweep across the lactase locus *LCT* [12],[32],[46] in CV but not in JW, in line with the fact that the latter is heterozygous for the European lactase advantageous mutation (Figure 1), while the former is homozygous. Third, we found that candidate genes detected empirically by our method (*K* averaged over the two individuals scanned independently) significantly overlap with those identified by alternative approaches [11]–[15], which include methods aimed at detecting partial or complete sweeps (Table 1; see Methods). Visual inspection of our data in comparison with previous scans (e.g. Williamson *et al.*, Carlson *et al.* and Pickrell *et al.*; see Methods) in the UCSC Genome Browser provides additional examples of convergent detection of positively selected loci by different methods (Table S3). Fourth, genes located in candidate sweeps tend to be more strongly expressed in cerebellum, spleen and testes comparatively to their expression in other tissues [47] (Figure S4), and generally *K* is significantly lower for several Gene Ontology biological processes [48],[49] already highlighted in previous scans for selective sweeps such as defence response or transcription [12],[16],[50] (Table S4). Therefore, despite the lower specificity and sensitivity expected from using data from individual genomes, the set of candidate sweeps detected by this method are highly enriched in positively selected loci (Text S2).

**Table 1:** Overlap of the current scan with published scans for selective sweeps.

	current scan	Williamson et al.	Voight et al.	Carlson et al.	Tang et al.	Pickrell et al.
current scan	787	67 (3.57) 3.10^−5^	49 (2.52) 2.10^−4^	178 (4.26) <10^−5^	68 (1.56) 2.10^−2^	61 (3.06) 6.10^−5^
Williamson et al.		444	41 (3.72) 6.10^−4^	217 (9.16) <10^−5^	83 (3.37) 10^−4^	53 (4.33) <10^−5^
Voight et al.			460	71 (2.91) 2.10^−4^	132 (5.17) <10^−5^	96 (6.81) <10^−5^
Carlson et al.				986	180 (3.28) <10^−5^	114 (4.62) <10^−5^
Tang et al.					1,030	171 (6.55) <10^−5^
Pickrell et al.						516

Cells in the diagonal indicate for each analysis the total number of candidate genes, which were selected using relaxed criteria to favour sensitivity over specificity (see Methods). For each comparison, the number of overlapping genes, the enrichment factor (between parentheses) and enrichment p-values measured with our co-occurrence test are indicated (see Methods).

### Co-occurring sweeps between primates reveal recent positive selection hotspots

**Figure 2:**
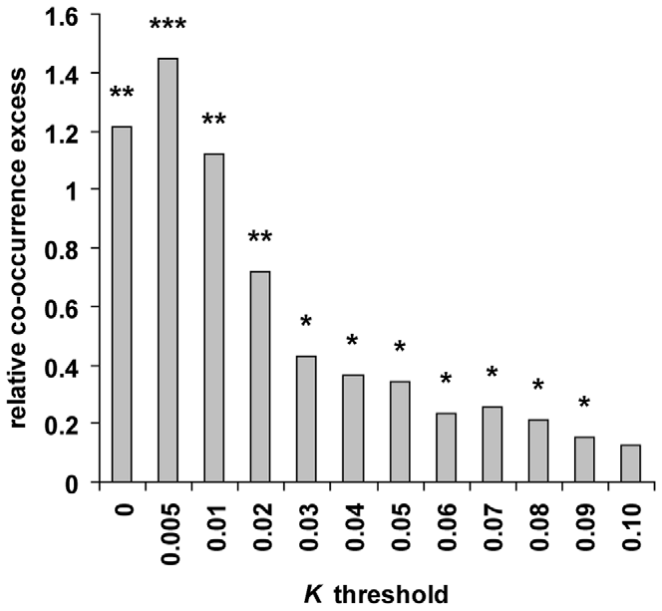
Human and other primate orthologous genes co-occur in selective sweeps more often than expected by chance. The relative co-occurrence excess (observed score/expected score –1) between human and other primate candidates was measured for several upper thresholds of *K* ranging from 0 to 0.1. *: *P*≤5.10^−2^. **: *P*≤10^−2^. ***: *P*≤10^−3^.

Our method is applicable to individual genomes and was used to scan the genomes of chimpanzee, orangutan and macaque that were all sequenced from single outbred individuals [51],[52]. We first used ssahaSNP2 [53] to identify 875,182, 1,364,646 and 2,294,239 heterozygous SNPs in chimpanzee, orangutan and macaque respectively (see Methods). In order to estimate how frequently recent positive selection has independently targeted the same gene in human and these other primate lineages, we computed the number of orthologous genes candidates for selection in human and at least one of the three other tested primates, and compared the results with random expectations. We first selected the 9,972 four-way protein coding orthologous genes tested in all primates, and identified candidate genes with *K* lower than threshold values ranging from 0 to 0.1 in human, chimpanzee, orangutan and macaque (see Methods; Figure 2 and Table S5). For each *K* threshold the number of candidates in non-human primates is higher than in human, because the use of two human individuals substantially increases specificity in this species and because non-human primate candidate genes are the sum of several scans with different window sizes. We devised a co-occurrence test that compares the observed numbers of genes found in candidate selective sweeps simultaneously in human and in one, two or three non-human primate species with the numbers expected if all genes are equally likely to experience positive selection (see Methods and Figure S5). The difference between observed and expected values thus reflects the excess or deficit of positive selection co-occurring at orthologous genes in multiple primates relative to the rate of positive selection in each primate. The ability of the test to detect an excess of co-occurrence, i.e. hotspots of positive selection, depends on three factors. The first is the “usage” frequency of a given hotspot by positive selection during evolution, which will affect the magnitude of the excess of co-occurrence. Indeed, hotspots might be used rarely enough that four species may not be sufficient to observe a significant excess. The second factor is the rate of false positive candidates in each tested species, which tend to occur randomly across genomes. False positives thus lead to underestimating the relative difference between real and random co-occurrences. However, we show that detecting hotspots of positive selection is possible even with a high rate of false positive candidates within each species (Texts S2, S3; Figure S6). The third factor is the power of the test to correctly identify, within each species, candidates for positive selection. Obviously, the lower the power the higher the risk of missing hotspots. For instance a hotspot active in three species where the power to detect positive selection events is only 30% will be identified with a power of only 2.7% (0.3^3^). Because the second and third factors act in opposite directions, the optimal *K* threshold to identify the footprint of hotspots through a statistically significant excess of co-occurring candidates of positive selection is therefore not the most stringent (few false positives but low power), but the one with the best compromise between power and the rate of false positives.

Using different *K* thresholds and controlling for several potentially confounding factors (see below), we find that although genes in candidate sweeps are mostly specific of a given primate, genes found in candidate sweeps in human and two or more other primates are systematically in significant excess (Figure 2 and Table S6). Overall, the relative co-occurrence excess increases with lower, more stringent *K* thresholds, as expected if false positives partly dissipate the signal of positive selection hotspots. Although we cannot precisely estimate their rate for different *K* values due to the approximate nature of population simulations, we expect the false positive rate to be high when using one or two individuals to detect sweeps, and most likely always above 50% of genes identified in selective sweeps (Text S2, Figures S1, S2, S3). Notably, the excess observed at the most stringent *K* = 0 is slightly lower than at the second most stringent *K*≤0.005, which likely reflects a loss of power to detect hotspots. Such a loss of power is also observed for the most stringent realisations of our test presented throughout this analysis (see below). Finally, we also analysed a set of 70 genes identified in common in at least three out of four previous human genome scans for selective sweeps [11]–[14] and thus likely to have a very low rate of false positives. Strikingly, 22 of the 70 genes (31%), nearly three times more than expected (11% expected, co-occurrence test *P* = 2.10^−3^; Table S7), are also in candidate sweeps in at least two non-human primates. These results are obtained by examining a short period of recent primate evolution and by comparing only four species with few individuals. We are therefore likely to underestimate the true frequency of positive selection hotspots active in human and other primates, which could plausibly be common and thus significantly impact the biological interpretations of human selective sweeps.

The level of co-occurrence could also be in principle the consequence of the presence of coldspots of positive selection instead of hotspots, where a fraction of genes are constantly under low rates of positive selection in all the lineages studied, leading to selective sweeps concentrating on the remaining genes. However, using a simple analytical model, we show that the level of co-occurrence observed here is most likely explained by hotspots of positive selection (Text S3 and Figure S6).

### Controlling for potentially confounding genomic factors

**Figure 3:**
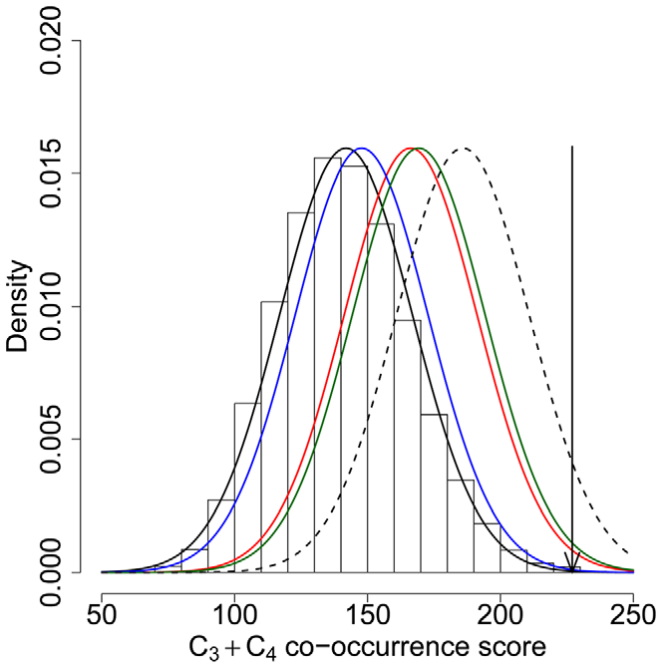
Orthologous genes co-occur in selective sweeps more often than expected by chance. There are 61 triplets and 11 quartets of orthologous genes that occur in putative selective sweeps simultaneously yet independently in human and two or three other primate genomes respectively (C_3_+C_4_ score = 227, black arrow). This is significantly more than expected by chance as shown by a co-occurrence test (black distribution) with 100,000 iterations, even after controlling for gene density alone (blue distribution), recombination alone (red distribution), or gene density and recombination simultaneously (green distribution) (see Methods). The dotted black distribution represents the hypothetical distribution where the observed score would still be significant at the 5% threshold.

Although false positives lead to underestimating the relative excess of co-occurring candidate genes, several genomic factors known to correlate with diversity could in contrast lead to overestimate the observed excess of co-occurrences. Such factors include sequencing depth, local divergence used in the estimation of *K*, base composition, gene density and recombination. As expected if our method correctly controls for sequencing heterogeneity, neutral mutation rate and composition (Text S1, Table S1), these factors explain a very small fraction of the variance of *K* (*n* = 18,605; human-chimpanzee divergence: Spearman's ρ = −0.015, *P* = 0.04; human-orangutan divergence: ρ = 0.026, *P* = 3.10^−4^; human-macaque divergence: ρ = 0.006, *P* = 0.43; human base composition: ρ = −0.014, *P* = 0.055; sequencing depth: ρ = −0.009, *P* = 0.19). In contrast, correlation of *K* is higher with gene density and recombination (*n* = 18,605; recombination rate: ρ = 0.18, *P*<10^−15^; gene density: ρ = −0.07, *P*<10^−15^). In order to quantify separately the effect of these factors on the relative excess of observed co-occurring candidate genes, we divided genes into ten classes of equal sizes according to the 10-quantiles of one specific factor, and ran separate randomizations in each class during the co-occurrence test (see Methods). Only gene density and recombination have a notable yet moderate impact on the expected level of co-occurrence, in agreement with correlations observed with *K* (Figure 3). The effect of recombination on co-occurrence supports findings that recombination rates tend to be conserved at a scale of 100 to 1,000 kb between closely related primates [54],[55]. We may however be exaggerating the effect of recombination in these controls, because the measures of recombination rates used here may be locally underestimated in the presence of selective sweeps [56]. As a consequence, low recombination classes tend to concentrate more candidate sweeps, thus leading to an overall inflated expected number of co-occurrences in the control. We nevertheless tested the impact of recombination and gene density simultaneously by further defining 100 different gene density/recombination combinations classes (see Methods). Although reduced, the relative excess of co-occurring candidates remains significant (Figure 2 and Figure 3).

We also tested the effect of using human-orangutan and chimpanzee-orangutan divergence instead of human-chimpanzee and chimpanzee-human divergence to compute *K* in human and chimpanzee, respectively. Measures of *K* with the two approaches show a 95% correlation coefficient, and more than 80% of genes are systematically below the same *K* threshold. None of the co-occurrence tests we conducted are affected, including the most compelling cases discussed below.

### Selective sweeps versus positive selection in coding sequences

**Figure 4:**
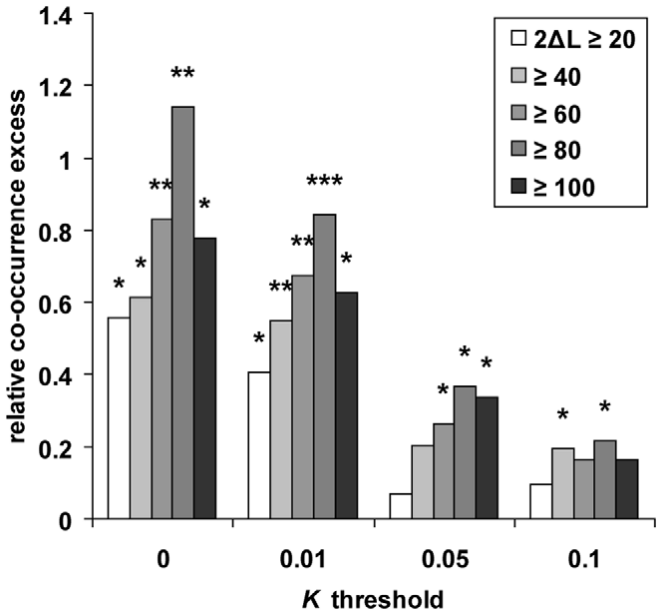
Recent human versus coding sequence positive selection. The co-occurrence test was used between coding sequence positive selection detected with the test 2 of PAML separately in five primate phylogenetic branches one the one hand, and recent positive selection in human and at least one additional primate one the other hand (see Methods; Figure S5). Genes were considered as positively selected in specific branches if twice the log-likelihood ratio (2ΔL) obtained with test 2 was greater than an arbitrary threshold comprised between 20 and 100. *: *P*≤5.10^−2^. **: *P*≤10^−2^. ***: *P*≤10^−3^.

If a fraction of genes with a low *K* in multiple primates represent positive selection hotspots, then those genes may have been positively selected not only during recent primate evolution, but also for longer evolutionary times. We therefore used the PAML branch-site likelihood ratio test 2 [20],[21] to analyse positive selection in orthologous coding sequences along five distinct branches of a phylogenetic tree including the four primates studied here, and using mouse as an outgroup [57] (see Methods). Using the co-occurrence test (Figure S5), we find a significant excess of co-occurrence between positive selection in coding sequences and thresholds of *K* ranging from 0 to 0.1 in human alone or human and at least one additional primate (Figure 4), thus extending our analysis to a much wider evolutionary time scale. Notably, the excess of co-occurrence increases substantially when using both more stringent *K* and more stringent inference of positive selection in coding sequences (with the exception of the most stringent conditions yielding slightly lower excess, again reflecting a plausible loss of power). Potentially biasing factors including recombination and gene density have no effect on this result. This therefore confirms positive selection hotspots independently from comparisons based only on recent selection, and shows that genes positively selected recently in human evolved similarly during more ancient primate evolution (Table S8).

### Non-human primates versus worldwide human populations

**Figure 5:**
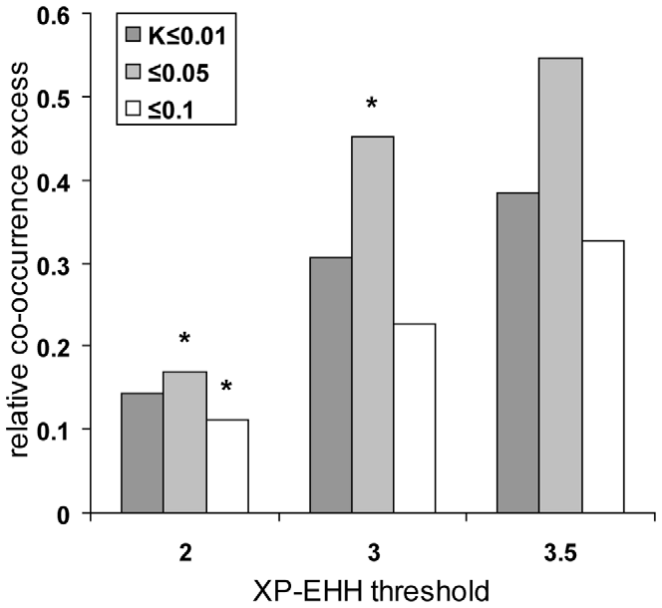
Co-occurrence between non-human primates selective sweep candidates and XP–EHH worldwide human populations selective sweep candidates. The co-occurrence test was applied using *K* in chimpanzee, orangutan and macaque to assign candidate genes on the one hand (Figure S5, group 1) and increasing XP-EHH values at genomic centers of genes tested in seven human populations on the other hand [5] (Figure S5, group 2). The histogram represents the relative co-occurrence excess obtained using 9,873 orthologous genes (observed score/expected score – 1) for XP-EHH values increasing from 2 to 3.5 with recombination and gene density being accounted for (see Methods). The excess of co-occurrence observed is lower for *K*≤0.01 than for *K≤*0.05, likely reflecting a loss of power to detect hotspots at the most stringent thresholds. *: co-occurrence test *P*≤0.05. **: *P*≤0.01. ***: *P*≤0.001.

In order to further validate the evidence for positive selection hotspots, we first compared the results obtained with our statistic *K* in chimpanzee, orangutan and macaque with recently published scans for selective sweeps in seven worldwide human populations using the XP-EHH test [15]. XP-EHH based scans of a representative set of human populations have several advantages over our own scan based only on two European individuals. First, XP-EHH shows a good power when identifying close to complete or recently completed selective sweeps even at low fixed false discovery rates [15],[17]. In line with this, XP-EHH has an excellent overlap with other scans in our comparison (Table 1). This should make the comparison of human with other primates more powerful despite a smaller absolute number of high confidence human candidates. Second, using seven human populations instead of one makes the comparison more representative. Using different *K* thresholds in non-human primates and increasingly high XP-EHH thresholds at genomic centres of human genes to isolate candidates, we confirm the previously observed excess of co-occurring selective sweeps candidates after controlling for recombination and gene density (Figure 5).

**Figure 6:**
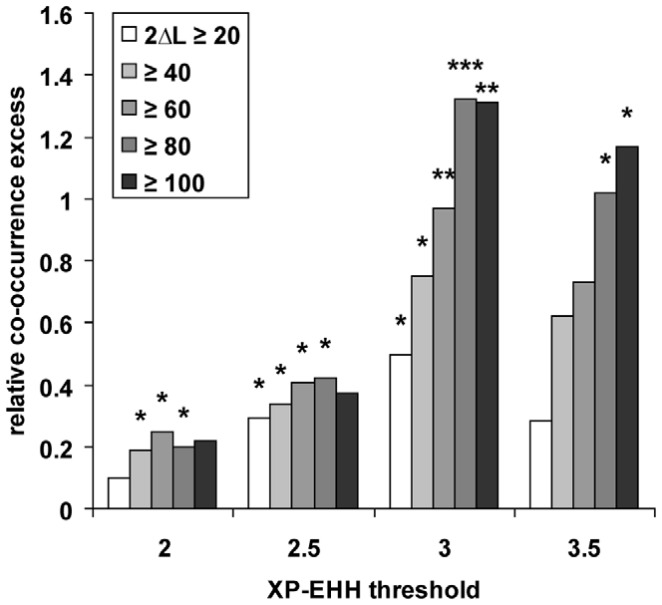
Co-occurrence between PAML branch-site test 2 positive selection candidates in non-human branches and XP-EHH worldwide human populations selective sweep candidates. The co-occurrence test was applied using increasing 2ΔL values (twice the log-likelihood ratio) to assign positive selection candidate genes in chimpanzee, orangutan, macaque and human-chimpanzee branches of a primate phylogenetic tree on the one hand (see Methods; Figure S5, group 1), and increasing XP-EHH values at genomic centres of genes tested in seven human populations [5] (Figure S5, group 2) on the other hand. The histogram represents the relative co-occurrence excess (observed/expected – 1) for combinations of XP-EHH threshold values increasing from 2 to 3.5 and branch-site test 2 2ΔL thresholds from 20 to 100 (Methods). *: co-occurrence test *P*≤0.05. **: *P*≤0.01. ***: *P*≤0.001.

In line with our previous observation that a comparison between recent (our test) and ancient (PAML's branch-site test 2) candidates for positive selection show an excess of co-occurring cases, we also find significant co-occurrence between worldwide human population XP-EHH candidates and PAML branch-site test 2 positive selection candidates in non-human branches of the primate tree (Figure 6). This last comparison further confirms the existence of “primate” positive selection hotspots recently active in human evolution. In particular, it does so independently of our own statistic *K*, and neither gene density nor recombination had an effect in this configuration of the co-occurrence test.

### Functional analysis of hotspot genes

The existence of positive selection hotspots is also supported by functional gene annotations. Several typical candidates of positive selection are over-represented among the functions of the 72 genes with *K*≤0.05 in human and at least two other primates, including defence response, gametogenesis and forebrain development (Table 2). Importantly however, these functions are represented alongside a wide variety of other biological processes and molecular functions, which cannot be due exclusively to false positives. Indeed overrepresented functions encompass only 2.1% of all GO terms included in hotspots, making it highly unlikely that these would concentrate all true positives. Recent positive selection hotspots are thus not limited to a few specific functions, but instead cover a diverse functional repertoire [58].

**Table 2:** Gene Ontology biological processes enriched in hotspots of recent positive selection.

GO biological process	number of genes	expected number	HGNC gene symbols	p-value
M phase (GO:0000279)	4	0.94	CUL7, CDC6, NCAPG, KLHDC3	0.01
defense response (GO:0006952)	6	1.86	AOC3, CAMLG, LONP1, TLR1, TLR6, TLR10	0.01
gametogenesis (GO:0007276)	5	0.95	SPA17, SPIN1, ACVR2A, SPATA2, TSSK3	0.002
spermatogenesis (GO:0007283)	4	0.74	SPA17, ACVR2A, SPATA2, TSSK3	0.006
forebrain development (GO:0030900)	3	0.17	TOP2B, FOXP2, HTRA2	0.0006

Significantly enriched Gene Ontology biological processes among the 72 candidate hotspots of recent positive selection in human and at least two other primates. The p-values and expected numbers are obtained with a randomization test (1 million repeats) based on annotations retrieved from FatiGO [48],[49] and were not corrected for multiple testing.

**Figure 7:**
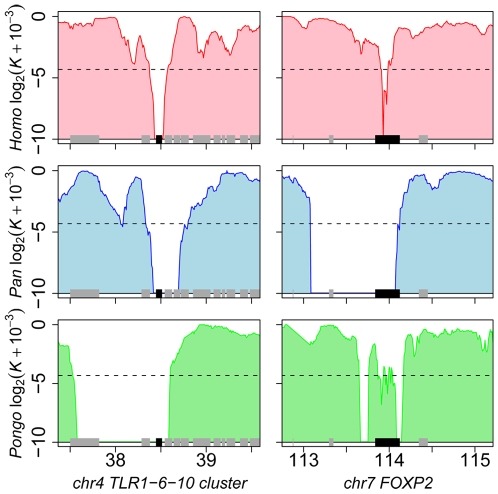
Candidate hotspots of recent positive selection at the Toll-like receptors 1, 6, and 10 cluster and the *FOXP2* locus. Each graph shows the variation of the log_2_ of *K* (+0.001 to avoid null values) at two candidate hotspots of recent adaptive evolution in human (average of the two individuals, red), chimpanzee (blue) and orangutan (green). To facilitate comparisons between genomes, values of *K* for chimpanzee and orangutan were projected on their human orthologous coordinates and gene symbols are those for human in all three species. Other legends are identical to Figure 1.

A finer inspection of candidate hotspots within over-represented functions reveals particularly interesting cases. Among defence response candidates, we infer independent selective sweeps in human, chimpanzee and orangutan for the cluster of Toll-like receptors (TLR) 1, 6 and 10 (Figure 7 and Figure S7). These receptors are involved in the non-specific recognition of a wide variety of bacteria during the first steps of the innate immune response. Interestingly, TLR 1, 6 and 10 are three of the nine strongest candidates with *K* = 0 in human, chimpanzee and orangutan in our analysis (Table S6), and were recently found as a strong case of local adaptation in the northern European population [15],[59], to which the two individuals used in the present study also belong. In addition, the role of TLR 1, 6 and 10 in the response to a broad spectrum of bacteria in multiple species is consistent with these receptors being hotspots of positive selection. Within gametogenesis *SPIN1* (Figure 7 and Figure S7) codes for spindlin, a protein involved in oocyte maturation [60]. *SPIN1* is one of the top ten candidates of the European population using the XP-EHH test [15], and is the strongest of our 13 strongest hotspot candidates (Table S6) with *K* = 0 in human, chimpanzee, orangutan and macaque, and is therefore a very strong positive selection hotspot candidate. The fact that several of the best candidates from the present analysis can be found within over-represented GO functions and, most importantly, are also found as top candidates in human using other methods argues strongly in favour of positive selection hotspots. Gametogenesis candidates also include the *ACVR2A* and *SPA17* (Figure 7) genes which both play a role during spermatogenesis [61],[62]. These examples show how multi-species comparisons may order priorities for deeper functional and evolutionary analyses among genes with positive selection during recent human evolution. More surprisingly, we found the *FOXP2* gene in candidate selective sweeps in human (*K* = 0.049), chimpanzee (*K* = 0) and orangutan (*K* = 0.049) (Figure 7 and Figure S7). *FOXP2* is an archetype of positively selected genes [43],[63] interpreted in the context of human-specific phenotypes, in this case linguistic processing. Yet our data suggests that positive selection on *FOXP2* recently occurred in other primates. Thus, recent positive selection on *FOXP2* may need to be also considered in the context of other *FOXP2* functions that could be shared among primates. This example further illustrates how interpretation of selective sweeps in human evolution can be guided by a broader and comparative view of positive selection among primates.

### Hotspots of positive or background selection?

Could co-occurring candidate genes be due to conserved background selection hotspots instead of recent positive selection hotspots [64],[65]? Although we cannot completely exclude an effect of background selection, our results are better explained by positive selection instead of recurrent deleterious mutations hotspots. First, functional analyses suggest that regions with low *K* in the human genome are dominated by positive selection. Indeed, several of the Gene Ontology biological processes found here with downwardly biased values of *K* were previously found over-represented for positively, not negatively selected genes [16]. Second, we find an excess of co-occurrence when comparing genes with *K*≤0.05 in non-human primates with positive selection candidates found in at least two of three human genome scans for selective sweeps that are insensitive to background selection [11],[12],[14] (relative co-occurrence score C_3_+C_4_ excess = 91%, co-occurrence test *P* = 7.10^−3^; Figure S5). Third, the co-occurrence between recent or coding sequence positive selection can be explained by positive, not background selection hotspots. Although background selection might have an influence, the presence of positive selection hotspots in primates active during recent human evolution remain the only reasonable explanation for our results.

## Discussion

We have performed a comparative analysis of positive selection in human and non-human primate genomes. Because non-human primate genomes do not benefit from genotyping data, we developed a new test to identify selective sweeps in single individual genomes. As shown by population simulations, this test is only moderately sensitive to demographic changes, and is thus widely applicable to genomes from single outbred individuals. The systematic comparison of genes positively selected during recent human evolution with candidates for positive selection in chimpanzee, orangutang and macaque shows a clear excess of genes that were positively selected independently in multiple primate lineages. This is independently confirmed by comparing recently published human positively selected genes [11]–[15] either to candidates identified by our test in non-human primates or to genes positively selected at the level of their coding sequence during more extensive evolutionary times. All these independent lines of evidence converge towards the same conclusion: primate genomes share hotspots of positive selection, including during recent human evolution.

Positive selection hotspots in primates raise several questions. First, do they mainly represent cases of parallel/convergent evolution, or cases of adaptations in different phenotypic directions involving the same locus? In the first scenario, hotspots could be seen as recurrent targets of positive selection for species under similar selective pressures. In the second scenario, hotspots would rather be considered as a toolbox to fine tune shared molecular functions according to different selective pressures. Second, we do not know how many genes can be described as positive selection hotspots in a primate genome. Each primate genome scan produced a high number of false positives, thus preventing unbiased attempts at estimating the minimal number of positive selection hotspots needed to explain our results. Third, the phylogenetic depth at which hotspots can be detected remains to be investigated. Our analysis in primates does not preclude that a subset of primate hotspots may be found under positive selection also in other mammals, or in other vertebrates. Indeed, isolated cases of parallel evolution have been observed between birds and rodents and human and fish [29],[31], raising the possibility that some hotspots may be shared among distant vertebrates.

A deeper knowledge of positive selection hotspots has additional practical and conceptual implications. Most scans for positively selected genes deliver false positive results [58],[66], thus making it difficult to interpret the evolution of any given gene in a biological context. Yet genes inferred to be positively selected independently in multiple scans or alternatively in multiple species within the same evolutionary period reduce this uncertainty because they are more likely to be true positives. More importantly, hotspots provide a means to identify positive selection events more specific to one species, and in particular positive selection events specific to human. For instance, 19 of the 70 human genes identified in common between at least three previous scans for selective sweeps [11]–[14] are not candidates in any of the three other primates, and are therefore more likely to be linked to specific human changes. For instance several of the 19 candidates belong to the same sweep as the lactase gene, an adaptive event associated with the emergence of cattle breeding and thus expected to be human-specific (Table S9). However, because our test and most of the published scans used for comparisons (except the scan of Voight *et al.*) aim at detecting complete sweeps, we cannot exclude that the most recent events of positive selection, those with still ongoing selective sweeps, reflect more specific human adaptation events.

We show here through independent comparisons based on a diverse array of methods that positive selection hotspots have been frequent during primate evolution, and in particular that genes positively selected during recent human evolution were also positively selected in other primate lineages, either recently or not. Yet importantly, our sampling of species and individuals is necessarily limited to sequenced genomes, and it is likely that the identification of hotspots of positive selection will increase in power as the genomes and variation information of more species become available. We predict that this will greatly facilitate the interpretation of biological changes underlying human selective sweeps. In particular, the identification of genes positively selected in human but never or infrequently in other primates will help to better outline truly specific aspects of recent human evolution. Our results provide a glimpse of the benefits of a hypothetical “1,000 Primate Genomes” project for the understanding of human adaptive evolution.

## Methods

### Estimation of *K*

We developed a new method inspired by the HKA test [36] to estimate the probability that a given locus has recently been subject to positive selection, using genome wide heterozygous sites and divergence data from single individuals. *K* values are computed for a given region *L* of size *q* (here 100 kb≤*q*≤300 kb) by first computing the ratio *r_l_* between the number of heterozygous sites and the number of divergent sites observed with a closely related species (e.g. human-chimpanzee when scanning the human and chimpanzee genomes, human-orangutan when scanning the orangutan genome and human-macaque when scanning the macaque genome). The same ratio *r_g_* is then computed for a region *G* extending 10 times *q* on both sides of *L*. A weighing scheme is applied to ratio *r_g_* to control for repeats, shotgun coverage and nucleotide composition (see next paragraph). The ratio *R_obs_* = *r_l_*/*r_g_* then expresses the local reduction (*R_obs_*<1) or increase (*R_obs_*>1) in heterozygosity given its level in the surrounding genomic region and the local divergence. Next, the ratio *R* is computed for 5,000 additional windows of size *q* randomly sampled within *G* but at a distance at least five times *q* from *L* (this is done with replacement, meaning that the same position in the background window can be represented in several random *q* sized windows). This generates an empirical distribution of *R* across the region *G*, thus providing an empirical means to estimate the probability of observing *R* lower than *R_obs_* in this region: *K* is the proportion of random windows with *R* lower than *R_obs_*. Because randomly sampled windows have globally comparable demographic histories, increases or decreases of the local diversity variance due to demographic expansions or contractions should be partially accounted for in the estimation of *K* (Text S2).

We introduced a weighing scheme to account for varying base composition and repeat density, two major factors that are known to affect genetic diversity in primate genomes. This scheme also controls for the random nature of genome shotgun sequencing, where variable numbers of reads covering a given position affect the probability of detecting the two alleles. To do so, we first compute *n_ij_* the number of positions of the tested window *L* occupied by a specific base *i* (A,T,C or G) identified or not in a repeat by RepeatMasker and covered by a number of reads *j*. For instance if a given window includes 4,500 positions occupied by nucleotide G outside of a repeat and covered by four reads then *n_G4_* = 4,500. The same procedure is applied for DNA inside repeats. Next, we compute *r_g_* for the genomic background window weighted by *n_ij_* in the tested window:
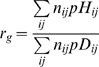
where *pH_ij_* and *pD_ij_* are respectively the proportions of heterozygous and divergent sites for all sites of class nucleotide *i* and coverage *j* in the genomic background window. We show, using simulations, that this weighing scheme removes the effect of a diverse range of factors that may bias measures of genetic diversity (Text S1). In this study, *K* was measured for local window sizes of 200 kb for human, 100, 150 and 200 kb for orangutan, 200 kb and 300 kb for chimpanzee, 70 kb, 100 kb and 140 kb for macaque. Larger windows were used for chimpanzee to account for its lower level of heterozygosity, while smaller windows were used for macaque due to its higher heterozygosity. The power to detect sweeps depends on the average initial level of heterozygosity (a lower initial level of heterozygosity means that there will be less contrast between a selective sweep and the neutral background), which can be compensated by adjusting window size so that the average number of heterozygous sites per window remains similar between species. Windows were excluded if they did not meet specific criterions: at least 60% of sites must be sequenced in the two species and be covered by less than 20 shotgun reads. For each measure of *K*, the genomic background window was adjusted to span 20 times the size of local windows, 10 times downstream and 10 times upstream. *K* was measured using 5,000 random resamplings for analyses with windows centred on genes (method validation and co-occurrence test), and 1,000 random samplings for windows sliding along chromosomes (Figure 1, Figure 7, Figure S7, Table S3).

### Co-occurrence test

The four primate species possess 14,480 mutual orthologs, of which 9,972 were tested in all four species. The remaining genes either reside on sexual chromosomes (human and chimpanzee genomes are sequenced from males, and thus provide no heterozygosity data for the X chromosome) or are located in windows that did not meet the required criterions to measure *K*. To test for co-occurrence, the following datasets were used for each species. In human, the average *K* between CV and JW was used to select candidate genes with *K* lower than a fixed threshold in 200 kb windows centred on the genomic centres of genes. These criterions showed the best overlap with published scans for selective sweeps [11]–[14]. In other primates, a candidate gene was selected if one of the three window sizes centred on this gene had *K* lower than the same threshold used for human. We then computed the sum of pairs (C_2_), triplets (C_3_) and quartets (C_4_) of genes seen in a putative sweep respectively in human and one, two or three species simultaneously (Figure S5). The human genome was then shuffled randomly and C_2_, C_3_ and C_4_ computed for 100,000 iterations. More specifically, for each iteration, the human genome was randomly divided into 20 intervals, with the gene order preserved within a given interval. Intervals were then rearranged in a random order and the sum C_2_, C_3_ and C_4_ computed across the randomized genome. The preservation of gene order within the intervals accounts for the clustered organisation of candidate sweep-associated orthologous genes. Clustering reflects the fact that a selective sweep in primate genomes often spans several neighbouring genes. This increases the variance, while leaving the average sum of co-occurrences unaffected. Increasing the number of intervals used for shuffling genes at each iteration did not change the results.

Several genomic factors such as recombination or gene density are correlated with *K* and have to be accounted for in our co-occurrence test. Such factors are indeed likely to increase the expected co-occurrence if they are conserved across species. We controlled for these factors separately by dividing the genes into *n* classes delimited by the *n−*1 quantiles of the factor to account for, and then running permutations within each class separately. We found that dividing the genes into 10 classes is sufficient in each case, since no gain of co-occurrence was observed when using more classes. Introducing classes however requires two corrections. First, dividing genes into classes can destroy clusters of contiguous candidate genes, thus reducing the variance of co-occurrence obtained after 100,000 permutations. Since the distribution of random co-occurrences is normal and since clustering does not affect the mean of this distribution but only its variance, we can address this issue by allocating the variance measured on the distribution without any class, to the distribution with 10 classes (Figure 3). Second, the simple fact of dividing genes into classes inflates the expected average level of co-occurrence in the presence of hotspots. This is due to the fact that each hotspot once “trapped” into a specific class will be randomized across a much smaller number of genomic locations and thus reconstructed randomly more frequently than when no class is defined. We accounted for this effect as follows: measures of *K* were first randomly permuted across genes, thus effectively removing the specific effect of any putative correlated genomic factors. This step was followed by 100 iterations of the co-occurrence test, and the two successive operations were repeated 1,000 times. The difference between the resulting average co-occurrence score and the average score when no classes are used finally represents the effect of using classes, independently of any genomic factor. This difference was therefore substracted from the average co-occurrence score every time classes were defined to account for genomic factors.

Finally, two factors could be tested simultaneously based on the information that a specific gene may for instance be in class 3 (out of 10) for factor 1 and in class 6 for factor 2, thus belonging to the class (3,6) used together with 99 other combinations in the co-occurrence test. This was done in particular to test the effect on co-occurrence of recombination and gene density considered simultaneously.

### Genome assemblies and alignments

Genome assemblies with softmasked repeated sequences identified by RepeatMasker, for human (HG18), chimpanzee (PanTro2), orangutan (PonAbe2) and macaque (RheMac2) were downloaded from the UCSC genome browser (http://hgdownload.cse.ucsc.edu/). Human-chimpanzee, chimpanzee-human, orangutan-human, human-orangutan, macaque-human and human-macaque Blastz alignments [67] in axt.net format were also downloaded from the UCSC genome browser.

### Coverage information

Levels of shotgun sequencing coverage were measured using different strategies depending on the availability of read location information. For chimpanzee, orangutan and macaque, coverage was directly deduced from read positions downloaded from the Washington University Genome Sequencing Center WUGSC website (http://genome.wustl.edu/pub/organism/Primates/) in reads.placed files. For the two human individuals, reads were first downloaded from the NCBI Trace archive site (ftp://ftp.ncbi.nih.gov/pub/TraceDB/) and mapped on the NCBI36 human genome assembly using Blat [68] with the -fastMap and minimal 95% identity options activated. Only those reads mapped on more than 80% of their length were retained to measure coverage.

### Heterozygous SNP detection

Heterozygous sites for the two human individuals were retrieved from the J. Craig Venter Institute web site (http://www.jcvi.org/) and from the Jim Watson Sequence website at CSHL (ftp://jimwatsonsequence.cshl.edu/jimwatsonsequence/), respectively. For chimpanzee, orangutan and macaque reads were first downloaded from the NCBI Trace archive (ftp://ftp.ncbi.nih.gov/pub/TraceDB/). Reads were then mapped on genome assemblies using ssahaSNP2 [53] (parsing parameters -identity = 92 -match = 80 -copy = 20 -cover = 20) to detect heterozygous SNPs.

### Gene annotations and orthology relationships

All analyses were conducted using Ensembl v48 annotations for protein coding genes and their homology relationships, except for PAML analysis where Ensembl v52 were used [69] (http://www.ensembl.org/). A total of 14,480 human-chimpanzee-orangutan-macaque four-way orthologs were found.

### PAML analysis

We used the likelihood ratio test 2 of the PAML package [20],[21] to detect positive selection separately in the five # labeled branches of the following phylogenetic tree:

(((human #, chimp #) #, orangutan #), macaque #, mouse)

We first retrieved protein and coding sequences of all Ensembl v52 human-chimp-orangutan-macaque-mouse five-way one-to-one orthologs. When a gene had multiple protein and coding sequences, only the longest were considered for further analysis. Nucleotides in chimpanzee, orangutan and macaque coding sequences with a Phred quality lower than 20 were excluded together with their 10 downstream and 10 upstream nucleotides neighbours. Downstream and upstream positions were also excluded because we noticed that nucleotides with quality lower than 20 were often found close to each other, thus raising doubts about the quality of interspaced nucleotides. This procedure indeed reduced the rate of false positives due to sequence inconsistencies (data not shown). Protein sequences were aligned with MAFFT [70] with high accuracy options activated. Coding sequence alignments were then obtained by projection on protein alignments. Only those 11,293 alignments containing at least 50 codons with no excluded nucleotide, starting with a start codon in one of the species and with at least one synonymous substitution between each pair of species were finally tested for positive selection.

### Analysis of microarray expression and Gene Ontology annotations

Affymetrix Human Exon microarray expression data for eleven tissues (breast, cerebellum, heart, kidney, liver, muscle, pancreas, prostate, spleen, testis and thyroid) was downloaded from the UCSC genome browser database. Values of expression for each Ensembl gene correspond to the average deduced from all probesets mapping the exons of a gene. Expressions of human genes candidates for positive selection were compared with expressions of the remaining genes using the log of Relative Abundance [47]. Gene Ontology annotations [71] of biological processes were analysed using the FatiGO and FatiScan [48],[49] software available at http://babelomics.bioinfo.cipf.es/.

### Measures of recombination rates, gene densities, and other genomic factors

Recombination and gene densities were controlled for in our test of co-occurrence. Recombination rates from the HapMap release 22 build 36 and estimated by LDHat [33],[54] were downloaded at http://www.hapmap.org/. The average recombination rate (cM/Mb) was calculated for 200 kb windows centred on genes. Gene densities were measured as the number of genes present within 100 kb downstream and 100 kb upstream of every gene. Other sizes from 200 kb to 1 Mb and from 100 kb to 2 Mb were investigated for gene density and recombination, respectively, but 100 kb and 200 kb are the ones showing the strongest effects when testing co-occurrence, respectively. Average sequencing depth, proportion of divergent sites for every possible pair of species, and average GC content were measured for windows ranging from 100 to 1000 kb, none of which had an impact on co-occurrence. Correlations shown in Results are for 200 kb windows.

### Comparison with other published human genome-wide scans

We compared our set of candidate positively selected genes (*K*≤0.05) in human with those found in four published scans for selective sweeps in the European population [11]–[15]. To compare several sets of genes and measure the level of observed overlap versus the level of expected overlap, the numbers of genes involved in the comparison must be of the same order of magnitude and large enough to avoid exceedingly high variance in estimated overlap. For these reasons and when needed, we use relaxed criteria to include larger numbers of genes in a given set than provided in highly specific shortlists in the original publications. By doing so, the frequency of potential false positive might increase, but this makes our conclusions conservative since the objective is only to compare the sets of genes relative to each other. In the study by Voight et al. [12] the 460 selected genes overlap regions where at least 20 out of 50 SNPs show an |iHS|≥2 in the HapMap phase II data. In the analysis by Williamson et al. [11] 444 genes with an associated p-value lower than 10^−4^ were selected. The 1,030 genes from the Tang et al. study [14] are those found within the candidate genomic intervals provided as supporting material of this publication. The 986 genes selected from the Carlson et al. scan [13] are those found within the 200 largest areas of negative Tajima's *D* in the genome. XP-EHH values were downloaded from the UCSC Genome Browser [15]. 516 genes with XP-EHH ≥2 at their genomic centre were used for comparison with other scans.

## Supporting Information

Figure S1 Performance of the test under panmictic and European populations demographic models using one individual. Power (Y axis) versus false positive rate (X axis) of the test to detect selective sweeps using 200 kb windows and 20 fold greater genomic background windows sliding every 10 kb are represented for a panmictic population (blue dots and curves) and for a demographic model of the European population (red dots and curves). Power and false positive rates were measured for a range of *K* thresholds for adaptive mutations fixed between 0 and 2,500 generations before testing (line 1), 2,500 and 5,000 (line 2), 5,000 and 7,500 (line 3) and 7,500 and 10,000 generations after fixation (line 4). Left column: selection coefficient *s* = 0.01. Right column: *s* = 0.1. Curves are second order polynomials fitted to the data.
(0.13 MB TIF)

Figure S2 Performance of the test under panmictic and European populations demographic models using two individuals. Same as Figure S1.
(0.15 MB TIF)

Figure S3 Performance of the test under panmictic and European populations demographic models using 20 individuals. Same as Figure S1. No curves could be fitted to the data.
(0.12 MB TIF)

Figure S4 Expression patterns of human selective sweeps candidate genes. The distributions of the log of Relative Abundance (RA) [47] measured for eleven human tissues with Affymetrix Human Exon microarrays (see Methods) were compared between human selective sweeps candidate genes (left distribution; *K*≤0.05, 563 candidates with complete expression information) and all other genes that were tested (right distribution; *K*>0.05, 12,652 genes with complete expression information). Using the Relative Abundance instead of absolute intensities allows us to identify tissues where candidate genes are up-regulated when compared to their expression in other tissues. *: Mann-Whitney U test, one sided *P*≤0.05. **: *P*≤0.01. ***: *P*≤0.001.
(0.24 MB TIF)

Figure S5 Randomization strategy for testing co-occurrence between two groups. Orange circles: genes with *K*> fixed threshold in group 1. Red circles: genes with *K*≤ fixed threshold in group 1. Light blue circles: genes with *K*> fixed threshold in group 2. Blue circles: genes with *K*≤ fixed threshold in group 2. In our case, group 1 represents non-human primates and group 2 represents human.
(1.71 MB TIF)

Figure S6 Combinations of *α* and *f_n_* that are compatible with the observed excess of co-occurrence of positive selection. The plot presents combinations of *α*, the proportion of genes with a low rate of positive selection in our model, and *f_n_*, the rate of false positives in non-human primates in our model that are compatible with the observed excess of co-occurrence between candidate genes for positive selection (Text S3).
(0.10 MB TIF)

Figure S7 Human-chimpanzee-orangutan candidate hotspots of recent positive selection at five loci. Each graph of lines 1, 2, and 3 shows the variation of the log_2_ of *K* (+0.001 to avoid null values) at candidate hotspots of recent positive selection in human (average of the two individuals, red), chimpanzee (blue) and orangutan (green). All five candidates belong to sets of genes with over-represented functions in the Gene Ontology. To facilitate comparisons between genomes, values of *K* for chimpanzee and orangutan were projected on their human orthologous coordinates and gene symbols are those for human in all three species. Other legends for graphs of lines 1, 2 and 3 are identical to Figure 1. From graphs on line 4 to graphs on line 12 all values were measured within 200 kb windows sliding every 10 kb. Line 4: human Venter + Watson heterozygosity. Line 5: chimpanzee heterozygosity. Line 6: orangutan heterozygosity. Line 7: Human-chimpanzee divergence. Line 8: Human-orangutan divergence. Line 9: Human GC content. Line 10: human sequencing depth (Venter+Watson). Line 11: chimpanzee sequencing depth. Line 12: orangutan sequencing depth. Importantly, this figure clearly shows that the five hotspot candidates presented are due to drops of heterozygosity and not to local anomalies in the other data that were used to calculate *K*.
(0.79 MB TIF)

Table S1 Evaluation of the weighing scheme to correct for *K* biasing factors. The table shows the Spearman's rank correlations of *K* with diverse simulated biasing factors whether or not the weighing scheme is applied (Methods; Text S1). *F*(*n*) (*n* being null or a positive integer) gives the probability that a heterozygous site is detected depending on the function *F* and the value *n* taken by the factor. Factor shape gives the shape of the gamma distribution used to model factor values and scale gives the scale parameter. Factor *a* and *b* give the values so that the final values *n* of the biasing factor were obtained by multiplying values from the gamma distributions with *a* and adding *b*. Any negative resulting value was set to 0. Region shape gives the shape of the gamma distribution used to model region sizes, Region scale the scale parameter. Region *a* and Region *b* give the values so that the final region size values were obtained by multiplying sizes from the gamma distribution with *a* and finally adding *b*. Any size lower than 1 kb was set to 1 kb.
(0.04 MB DOC)

Table S2 Average *K* for 19,331 Ensembl human genes. Column 1: Ensembl gene ID. Column 2: chromosome. Columns 3 and 4: Gene start and end coordinates on NCBI36/HG18. Column 5: Average *K* for CV and JW for 200 kb windows centred on the genomic centre of each genes.
(0.85 MB TXT)

Table S3 Custom tracks for visualizing scan results in the UCSC Genome Browser. The wiggle file contains three custom tracks that can be visualized via upload in the UCSC Genome Browser. The first track represents the average *K* obtained from 1,000 random samplings for 200 kb windows sliding every 10 kb. Other tracks highlight regions with CLR *P*≤0.05, and CLR *P*≤10^−4^ remapped on the NCBI36/HG18 assembly in the Williamson et al. study [11]. Users can also activate the Tajima's *D*, XP-EHH and iHS tracks already available in the UCSC Genome Browser for further comparisons.
(4.08 MB TXT)

Table S4 Gene Ontology biological processes with significantly lower distributions of *K in the human genome*. Gene Ontology biological processes with lower values of *K* than the rest of the genome were identified using the FatiScan [48],[49] tool available at http://babelomics.bioinfo.cipf.es/. Several parent and daughter significant processes of those indicated were removed. FDR is for False Discovery Rate.
(0.03 MB DOC)

Table S5 Values of *K* for human, chimpanzee, orangutan and macaque used in the co-occurrence test. Column 1: Human Ensembl gene ID. Column 2: human chromosome. Columns 3 and 4: gene start and end in human. Columns 5, 6, 7, 8: *K* in human, chimpanzee, orangutan and macaque, respectively.
(0.62 MB TXT)

Table S6 Best candidate hotspots of recent positive selection. Column 1: HGNC symbol. Other columns are the same as for Table S5.
(0.05 MB DOC)

Table S7 Candidate genes for positive selection in three or four published scans [11]–[14] and in putative sweeps in two or more non-human primates.
(0.07 MB DOC)

Table S8 Top candidates identified in both the PAML test on coding sequences and our test for selective sweeps (recent positive selection).
(0.05 MB DOC)

Table S9 Ensembl genes observed in a selective sweep only in human. Note that all candidates on chromosome 2 except the first correspond to the lactase locus.
(0.05 MB DOC)

Text S1 Weighing scheme evaluation.
(0.02 MB DOC)

Text S2 Forward population simulations.
(0.03 MB DOC)

Text S3 Distinguishing between hotspots and coldspots models.
(0.03 MB DOC)
