# Supplementary figures and images for: Human and Non-Human Primate Genomes Share Hotspots of Positive Selection
Source: PLoS Genet. 2010 Feb 5;6(2):e1000840. doi: 10.1371/journal.pgen.1000840 (PMC2816677; doi:10.1371/journal.pgen.1000840)

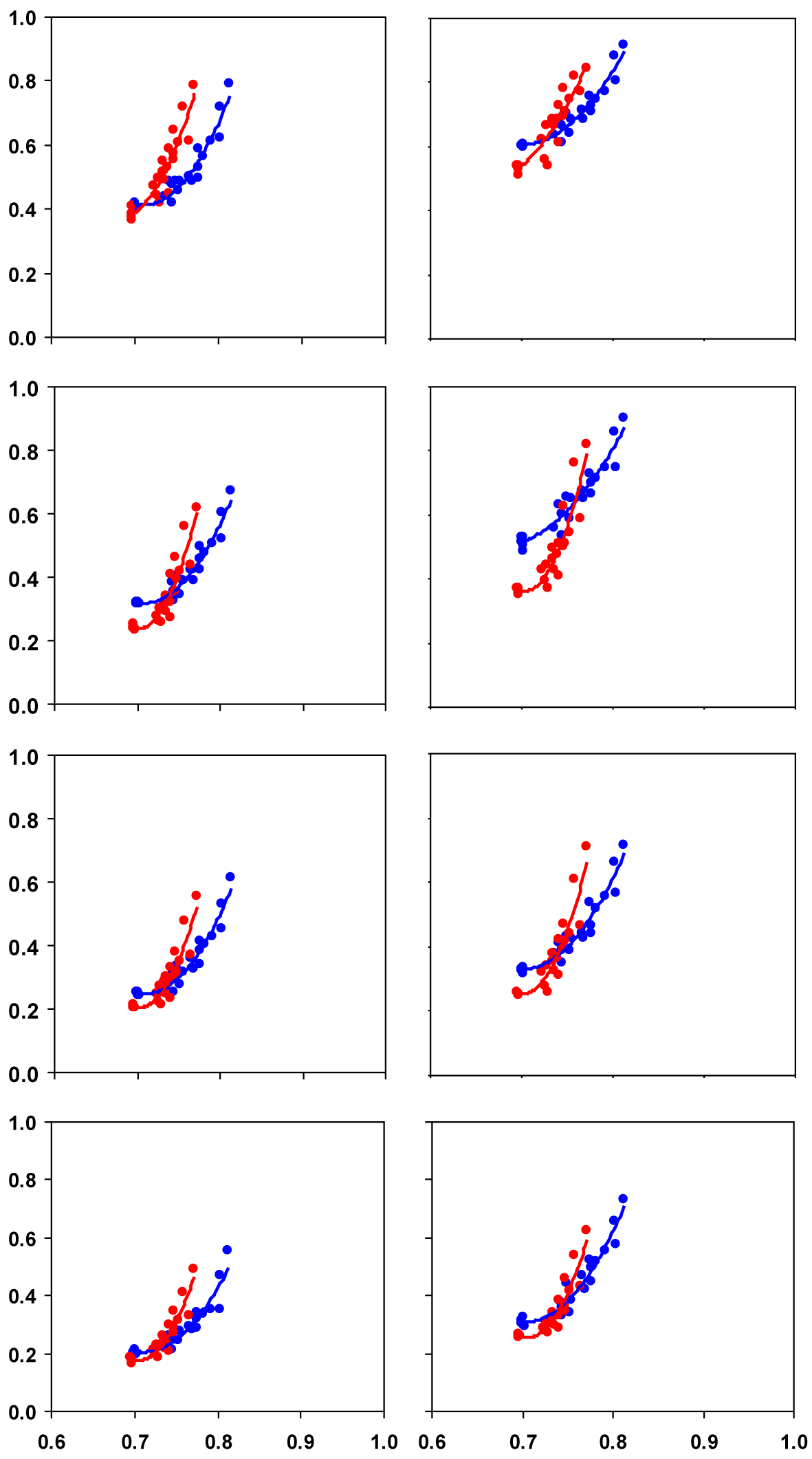

Supplement: Figure S1 — Performance of the test under panmictic and European populations demographic models using one individual. Power (Y axis) versus false positive rate (X axis) of the test to detect selective sweeps using 200 kb windows and 20 fold greater genomic background windows sliding every 10 kb are represented for a panmictic population (blue dots and curves) and for a demographic model of the European population (red dots and curves). Power and false positive rates were measured for a range of K thresholds for adaptive mutations fixed between 0 and 2,500 generations before testing (line 1), 2,500 and 5,000 (line 2), 5,000 and 7,500 (line 3) and 7,500 and 10,000 generations after fixation (line 4). Left column: selection coefficient s = 0.01. Right column: s = 0.1. Curves are second order polynomials fitted to the data. (0.13 MB TIF) [file pgen.1000840.s001.tif]

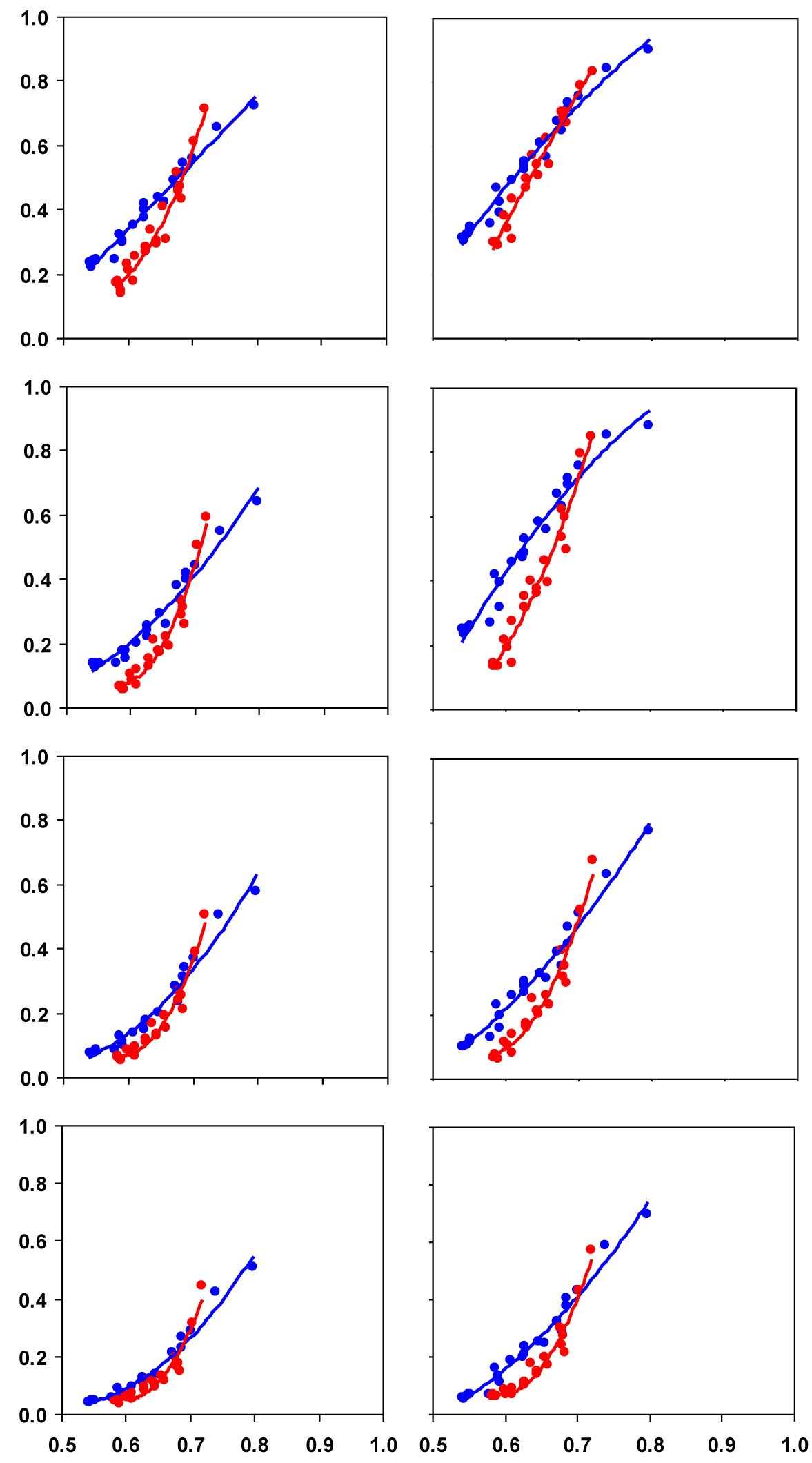

Supplement: Figure S2 — Performance of the test under panmictic and European populations demographic models using two individuals. Same as Figure S1. (0.15 MB TIF) [file pgen.1000840.s002.tif]

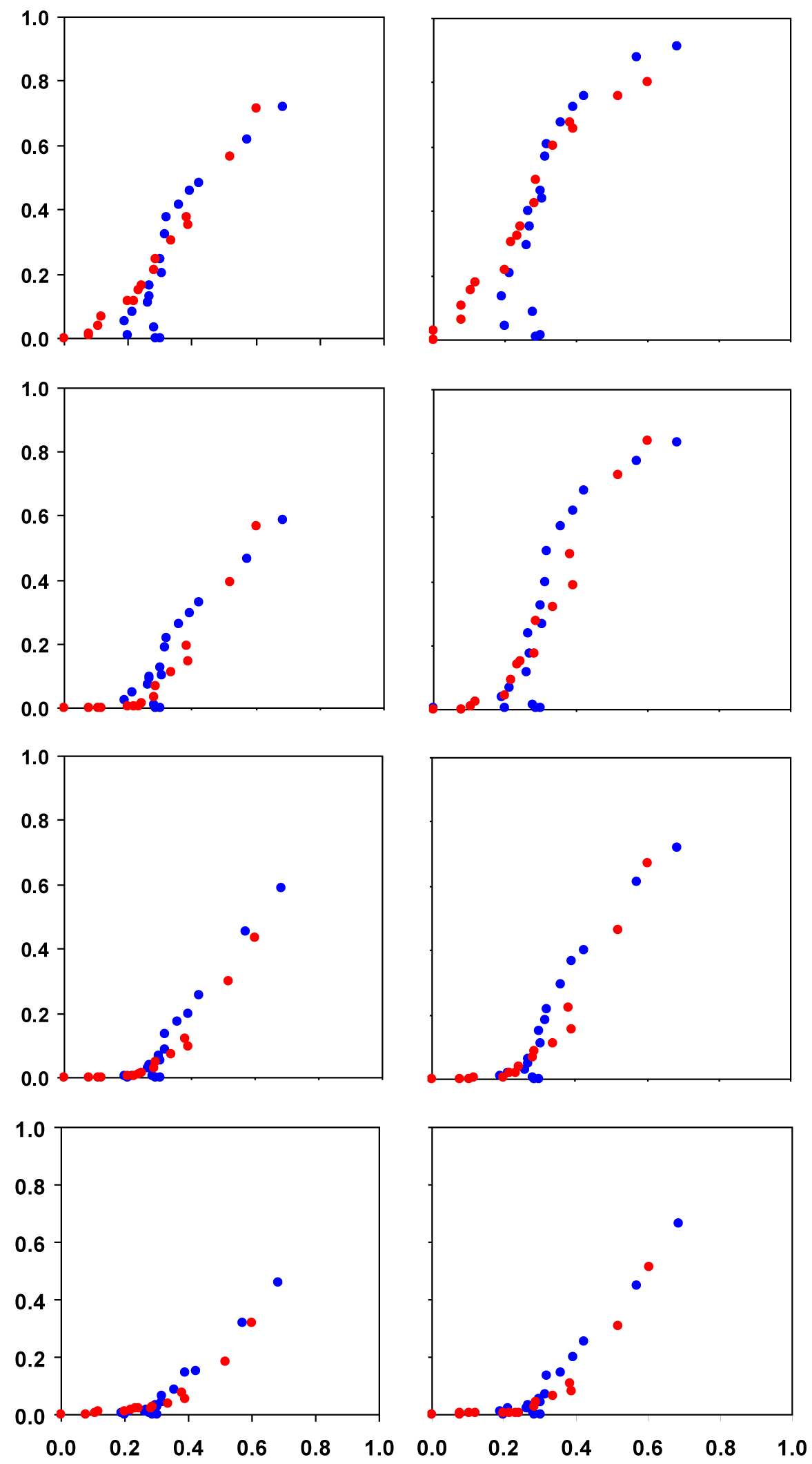

Supplement: Figure S3 — Performance of the test under panmictic and European populations demographic models using 20 individuals. Same as Figure S1. No curves could be fitted to the data. (0.12 MB TIF) [file pgen.1000840.s003.tif]

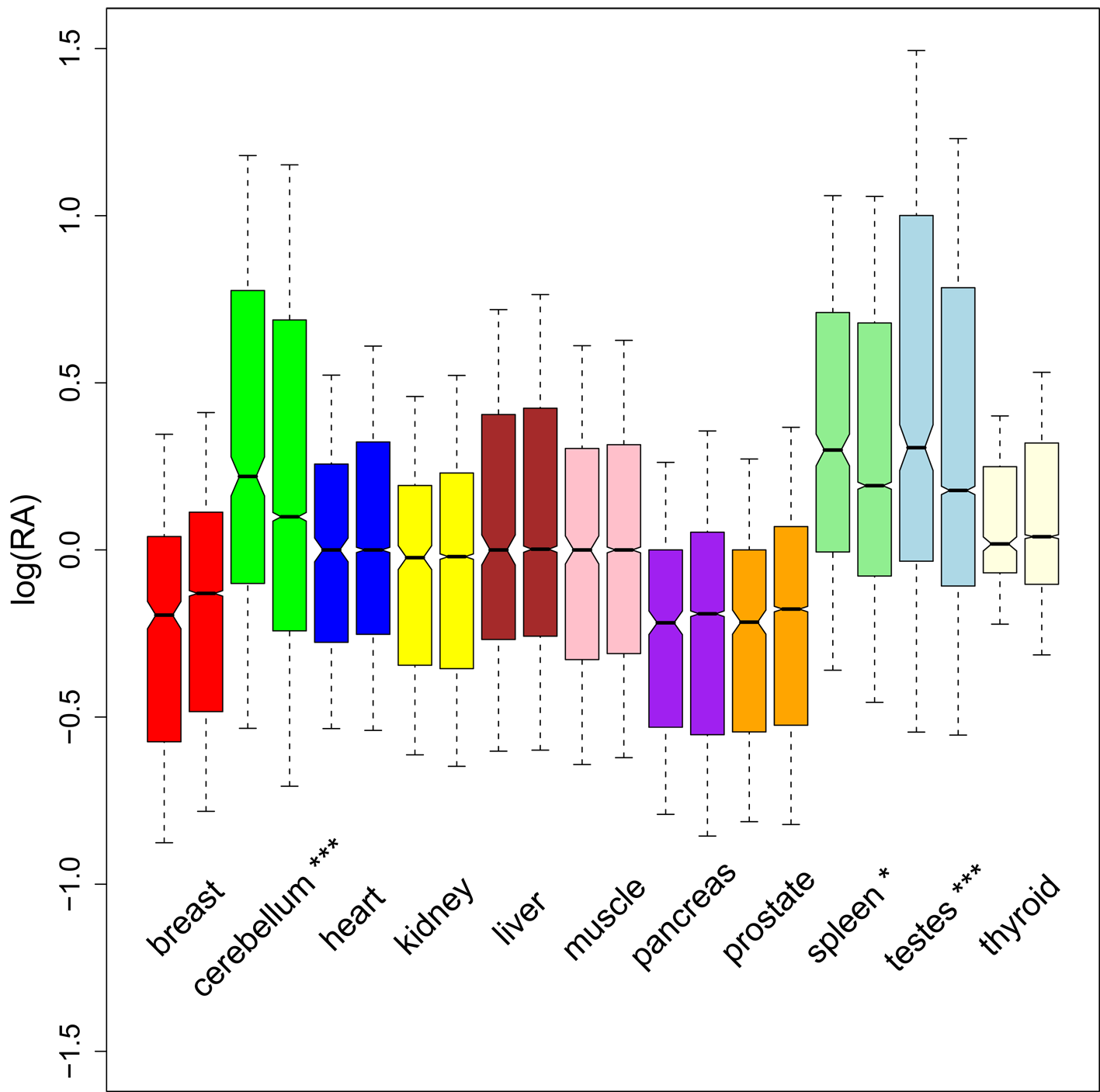

Supplement: Figure S4 — Expression patterns of human selective sweeps candidate genes. The distributions of the log of Relative Abundance (RA) [47] measured for eleven human tissues with Affymetrix Human Exon microarrays (see Methods) were compared between human selective sweeps candidate genes (left distribution; K≤0.05, 563 candidates with complete expression information) and all other genes that were tested (right distribution; K>0.05, 12,652 genes with complete expression information). Using the Relative Abundance instead of absolute intensities allows us to identify tissues where candidate genes are up-regulated when compared to their expression in other tissues. *: Mann-Whitney U test, one sided P≤0.05. **: P≤0.01. ***: P≤0.001. (0.24 MB TIF) [file pgen.1000840.s004.tif]

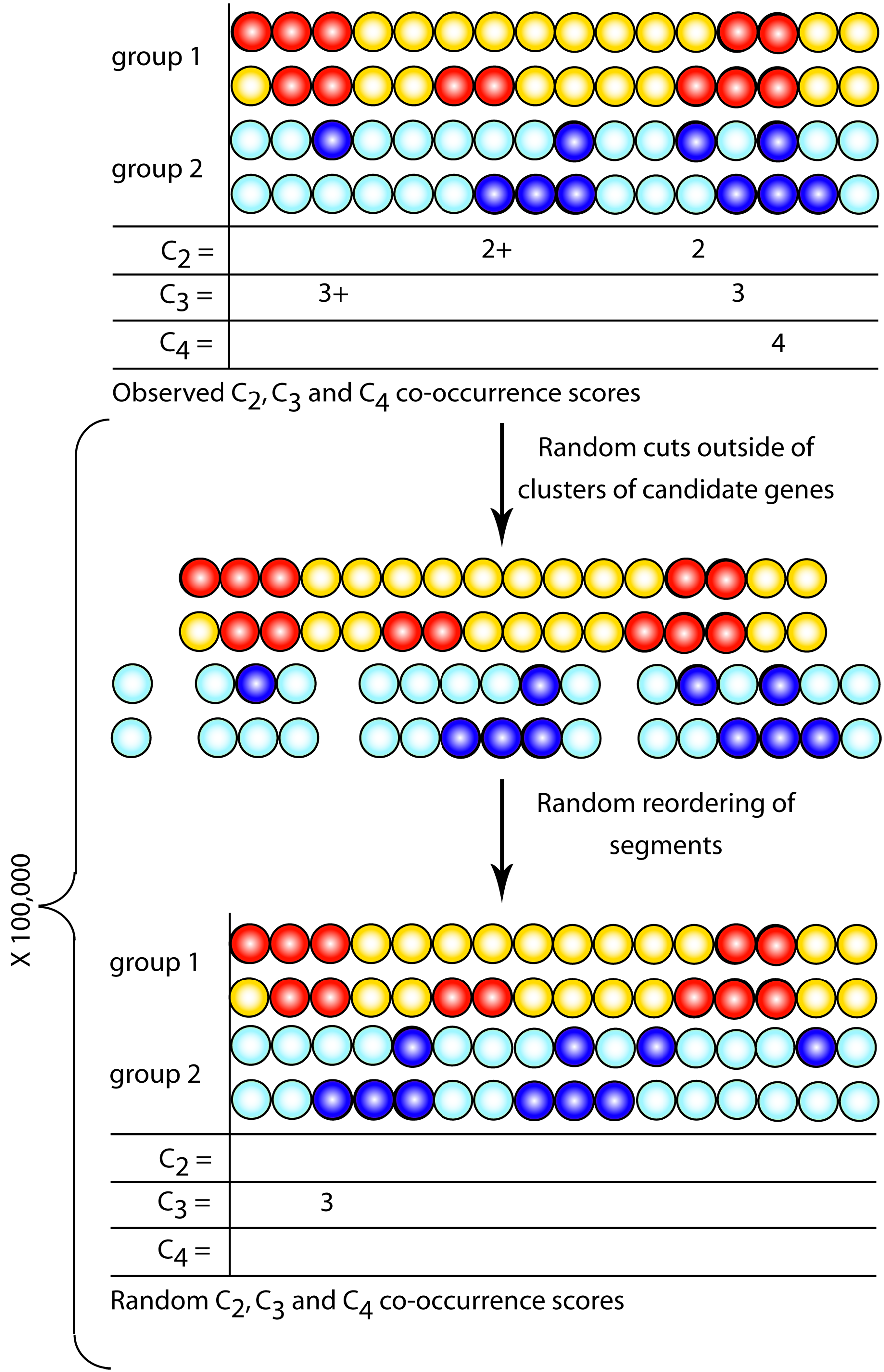

Supplement: Figure S5 — Randomization strategy for testing co-occurrence between two groups. Orange circles: genes with K> fixed threshold in group 1. Red circles: genes with K≤ fixed threshold in group 1. Light blue circles: genes with K> fixed threshold in group 2. Blue circles: genes with K≤ fixed threshold in group 2. In our case, group 1 represents non-human primates and group 2 represents human. (1.71 MB TIF) [file pgen.1000840.s005.tif]

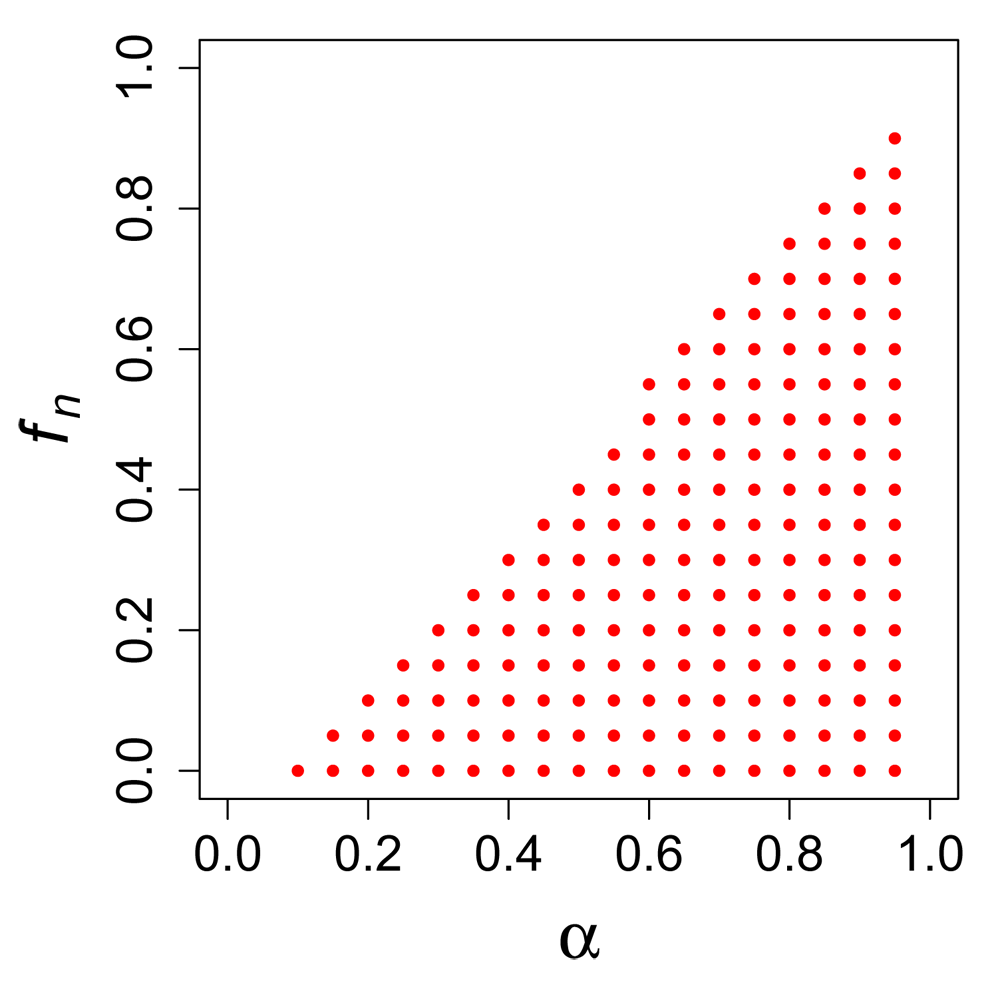

Supplement: Figure S6 — Combinations of α and fn that are compatible with the observed excess of co-occurrence of positive selection. The plot presents combinations of α, the proportion of genes with a low rate of positive selection in our model, and fn, the rate of false positives in non-human primates in our model that are compatible with the observed excess of co-occurrence between candidate genes for positive selection (Text S3). (0.10 MB TIF) [file pgen.1000840.s006.tif]

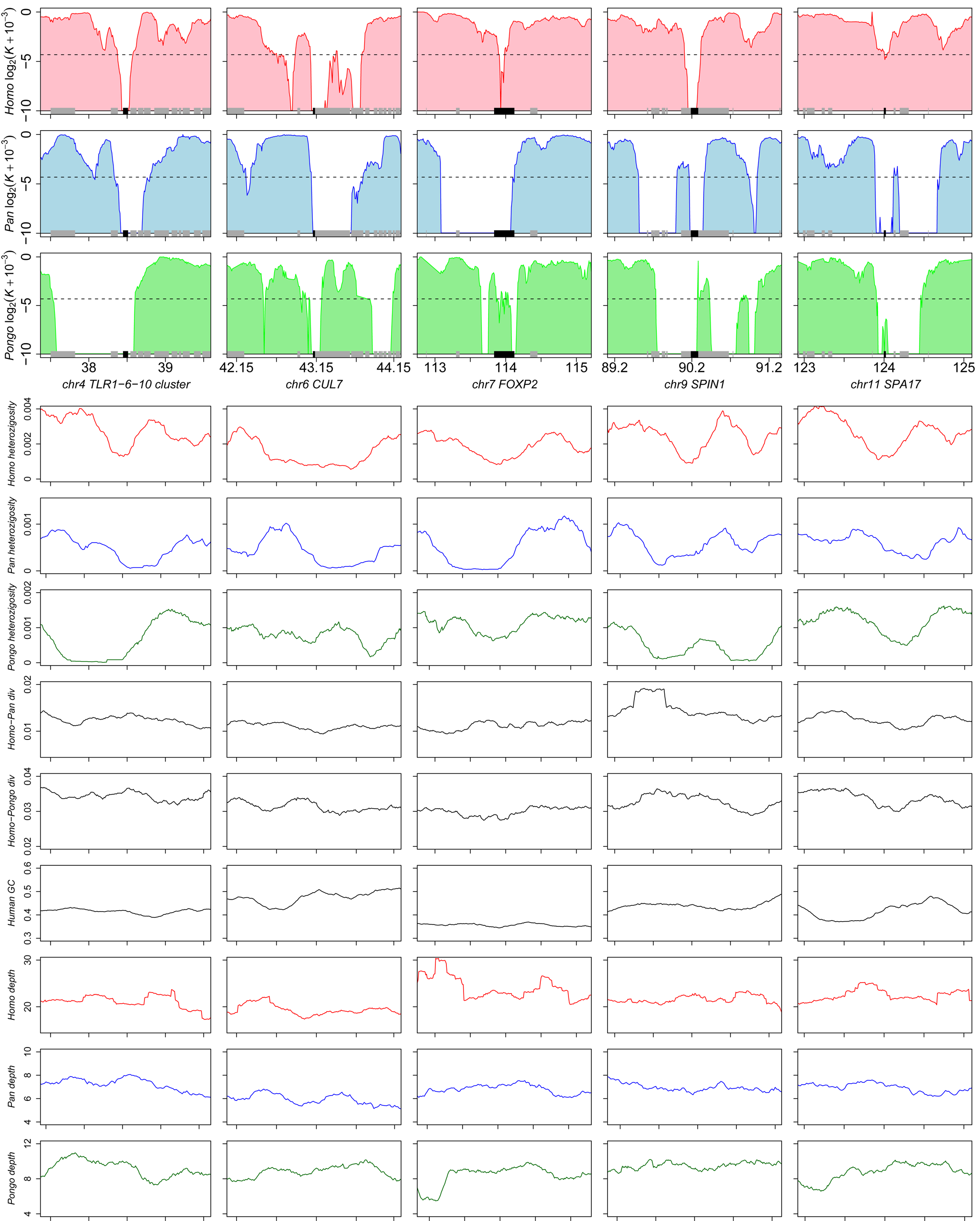

Supplement: Figure S7 — Human-chimpanzee-orangutan candidate hotspots of recent positive selection at five loci. Each graph of lines 1, 2, and 3 shows the variation of the log2 of K (+0.001 to avoid null values) at candidate hotspots of recent positive selection in human (average of the two individuals, red), chimpanzee (blue) and orangutan (green). All five candidates belong to sets of genes with over-represented functions in the Gene Ontology. To facilitate comparisons between genomes, values of K for chimpanzee and orangutan were projected on their human orthologous coordinates and gene symbols are those for human in all three species. Other legends for graphs of lines 1, 2 and 3 are identical to Figure 1. From graphs on line 4 to graphs on line 12 all values were measured within 200 kb windows sliding every 10 kb. Line 4: human Venter + Watson heterozygosity. Line 5: chimpanzee heterozygosity. Line 6: orangutan heterozygosity. Line 7: Human-chimpanzee divergence. Line 8: Human-orangutan divergence. Line 9: Human GC content. Line 10: human sequencing depth (Venter+Watson). Line 11: chimpanzee sequencing depth. Line 12: orangutan sequencing depth. Importantly, this figure clearly shows that the five hotspot candidates presented are due to drops of heterozygosity and not to local anomalies in the other data that were used to calculate K. (0.79 MB TIF) [file pgen.1000840.s007.tif]
