# Supplementary material for: Human and Non-Human Primate Genomes Share Hotspots of Positive Selection
Source: PLoS Genet. 2010 Feb 5;6(2):e1000840. doi: 10.1371/journal.pgen.1000840 (PMC2816677; doi:10.1371/journal.pgen.1000840)
Supplement: Table S1 — Evaluation of the weighing scheme to correct for K biasing factors. The table shows the Spearman's rank correlations of K with diverse simulated biasing factors whether or not the weighing scheme is applied (Methods; Text S1). F(n) (n being null or a positive integer) gives the probability that a heterozygous site is detected depending on the function F and the value n taken by the factor. Factor shape gives the shape of the gamma distribution used to model factor values and scale gives the scale parameter. Factor a and b give the values so that the final values n of the biasing factor were obtained by multiplying values from the gamma distributions with a and adding b. Any negative resulting value was set to 0. Region shape gives the shape of the gamma distribution used to model region sizes, Region scale the scale parameter. Region a and Region b give the values so that the final region size values were obtained by multiplying sizes from the gamma distribution with a and finally adding b. Any size lower than 1 kb was set to 1 kb. (0.04 MB DOC) [file pgen.1000840.s008.doc]

| SNP calling probability | Factor shape, scale, a, b | Region shape, scale, a, b | ρ | *P* | ρ corrected | *P* corrected | factor mean |
| --- | --- | --- | --- | --- | --- | --- | --- |
| *F*(*n*) = 1 - 2(0.5)*^n^* | 1.5, 10, 10^-3^, 0 | 2, 5.10^3^, 1, 0 | 0.09 | < 10^-16^ | 0.00 | 9.5.10^-1^ | 7.67 |
|  | 1.5, 10, -10^-3^, 10 | 1.5, 10^4^, -1, 5.10^4^ | 0.15 | < 10^-16^ | -0.01 | 9.10^-4^ | 3.67 |
|  | 0.5, 1, 7.10^-2^, 0 | 0.5, 5.10^4^, 1, 0 | -0.03 | < 10^-16^ | -0.02 | 4.1.10^-8^ | 5.11 |
| *n*<10, *F*(*n*) = 0.1*n* | 1.5, 10, 10^-3^, 0 | 2, 5.10^3^, 1, 0 | 0.14 | < 10^-16^ | -0.01 | 6.1.10^-3^ | 5.49 |
| *n*≥10, *F*(*n*) = 1 | 1.5, 10, -10^-3^, 10 | 1.5, 10^4^, -1, 5.10^4^ | 0.14 | < 10^-16^ | 0.01 | 1.8.10^-6^ | 5.51 |
|  | 0.5, 1, 7.10^-2^, 0 | 0.5, 5.10^4^, 1, 0 | 0.42 | < 10^-16^ | -0.02 | 4.4.10^-9^ | 5.33 |
