# Supplementary material for: Human and Non-Human Primate Genomes Share Hotspots of Positive Selection
Source: PLoS Genet. 2010 Feb 5;6(2):e1000840. doi: 10.1371/journal.pgen.1000840 (PMC2816677; doi:10.1371/journal.pgen.1000840)
Supplement: Table S4 — Gene Ontology biological processes with significantly lower distributions of K in the human genome. Gene Ontology biological processes with lower values of K than the rest of the genome were identified using the FatiScan [48],[49] tool available at http://babelomics.bioinfo.cipf.es/. Several parent and daughter significant processes of those indicated were removed. FDR is for False Discovery Rate. (0.03 MB DOC) [file pgen.1000840.s011.doc]

| GO biological process | p-value | FDR adjusted p-value |
| --- | --- | --- |
| response to DNA damage stimulus (GO:0006974) | 8.5.10^-6^ | 9.1.10^-4^ |
| chromatin assembly or disassembly (GO:0006333) | 4.9.10^-6^ | 2.3.10^-3^ |
| DNA replication (GO:0006260) | 3.2.10^-6^ | 1.9.10^-3^ |
| regulation of cell cycle (GO:0051726) | 7.9.10^-5^ | 8.1.10^-3^ |
| transcription (GO:0006350) | 4.3.10^-5^ | 5.9.10^-3^ |
| response to other organism (GO:0051707) | 1.2.10^-4^ | 5.3.10^-3^ |
| defence response (GO:0006952) | 6.5.10^-4^ | 8.2.10^-3^ |
| cytokine production (GO:0001816) | 3.1.10^-4^ | 4.6.10^-3^ |
| leukocyte activation (GO:0045321) | 1.2.10^-3^ | 3.4.10^-2^ |
