# Supplementary material for: Human and Non-Human Primate Genomes Share Hotspots of Positive Selection
Source: PLoS Genet. 2010 Feb 5;6(2):e1000840. doi: 10.1371/journal.pgen.1000840 (PMC2816677; doi:10.1371/journal.pgen.1000840)
Supplement: Table S6 — Best candidate hotspots of recent positive selection. Column 1: HGNC symbol. Other columns are the same as for Table S5. (0.05 MB DOC) [file pgen.1000840.s013.doc]

| HGNC symbol | Ensembl ID | chr | start | end | Human *K* | Pan *K* | Pongo *K* | Macaque *K* |
| --- | --- | --- | --- | --- | --- | --- | --- | --- |
| CCDC142 | ENSG00000135637 | 2 | 74554737 | 74563631 | 0 | 0 | 0 | 0.0114 |
| TTC31 | ENSG00000115282 | 2 | 74563718 | 74575191 | 0 | 0.01 | 0 | 0 |
| PCGF1 | ENSG00000115289 | 2 | 74585678 | 74589215 | 0 | 0.0032 | 0 | 0 |
| TLR10 | ENSG00000174123 | 4 | 38450658 | 38460984 | 0 | 0 | 0 | 0.7508 |
| TLR1 | ENSG00000174125 | 4 | 38474275 | 38482807 | 0 | 0 | 0 | 0.879 |
| TLR6 | ENSG00000174130 | 4 | 38504618 | 38507555 | 0 | 0 | 0 | 0.9196 |
| C6ORF153 | ENSG00000124541 | 6 | 43097351 | 43105312 | 0 | 0 | 0 | 0.3918 |
| CUL7 | ENSG00000044090 | 6 | 43113336 | 43129632 | 0 | 0 | 0 | 0.4116 |
| KLC4 | ENSG00000137171 | 6 | 43135350 | 43150815 | 0 | 0 | 0 | 0.385 |
| PTK7 | ENSG00000112655 | 6 | 43152007 | 43237435 | 0 | 0 | 0 | 0.336 |
| GARS | ENSG00000106105 | 7 | 30600706 | 30640168 | 0 | 0.391 | 0 | 0 |
| SPIN1 | ENSG00000106723 | 9 | 90193117 | 90283443 | 0 | 0 | 0 | 0 |
| RFX2 | ENSG00000087903 | 19 | 5944175 | 6061554 | 0 | 0.7926 | 0 | 0 |
