# Supplementary material for: Human and Non-Human Primate Genomes Share Hotspots of Positive Selection
Source: PLoS Genet. 2010 Feb 5;6(2):e1000840. doi: 10.1371/journal.pgen.1000840 (PMC2816677; doi:10.1371/journal.pgen.1000840)
Supplement: Table S7 — Candidate genes for positive selection in three or four published scans [11]–[14] and in putative sweeps in two or more non-human primates. (0.07 MB DOC) [file pgen.1000840.s014.doc]

| Ensembl gene ID | chr | start | end | Pan *K* | Pongo *K* | Macaque *K* |
| --- | --- | --- | --- | --- | --- | --- |
| ENSG00000005448 | 2 | 74502365 | 74506389 | 0 | 0 | 0.4252 |
| ENSG00000114993 | 2 | 74506497 | 74522568 | 0 | 0 | 0.433 |
| ENSG00000115274 | 2 | 74535707 | 74541526 | 0 | 0 | 0.0816 |
| ENSG00000159239 | 2 | 74494964 | 74496885 | 0 | 0 | 0.488 |
| ENSG00000115275 | 2 | 74541712 | 74546026 | 0 | 0 | 0.0754 |
| ENSG00000135637 | 2 | 74554737 | 74563631 | 0 | 0 | 0.0114 |
| ENSG00000115282 | 2 | 74563718 | 74575191 | 0.01 | 0 | 0 |
| ENSG00000115289 | 2 | 74585678 | 74589215 | 0.0032 | 0 | 0 |
| ENSG00000115297 | 2 | 74595119 | 74597396 | 0.0026 | 0 | 0 |
| ENSG00000144045 | 2 | 74598766 | 74606826 | 0.0028 | 0 | 0 |
| ENSG00000115307 | 2 | 74607284 | 74610482 | 0.005 | 0 | 0.007 |
| ENSG00000115317 | 2 | 74610040 | 74614191 | 0.0018 | 0 | 0 |
| ENSG00000115318 | 2 | 74613454 | 74634570 | 0 | 0 | 0.0526 |
| ENSG00000115325 | 2 | 74634795 | 74638181 | 0 | 0 | 0.1104 |
| ENSG00000159374 | 2 | 74638527 | 74728672 | 0.0432 | 0 | 0.434 |
| ENSG00000135622 | 2 | 747340901 | 74762693 | 0.0346 | 0 | 0.346 |
| ENSG00000138669 | 4 | 82228861 | 82355212 | 0.8264 | 0 | 0.0156 |
| ENSG00000186952 | 5 | 109931539 | 109989052 | 0.9668 | 0 | 0.0336 |
| ENSG00000164209 | 5 | 110102653 | 110126381 | 0.0428 | 0 | 0.0352 |
| ENSG00000182645 | 10 | 118073930 | 118129530 | 0.0158 | 0 | 0.6548 |
| ENSG00000027075 | 14 | 60858186 | 61087443 | 0 | 0.3056 | 0.0012 |
| ENSG00000154001 | 14 | 62911108 | 63079832 | 0.0228 | 0.2734 | 0 |
