# Supplementary material for: Human and Non-Human Primate Genomes Share Hotspots of Positive Selection
Source: PLoS Genet. 2010 Feb 5;6(2):e1000840. doi: 10.1371/journal.pgen.1000840 (PMC2816677; doi:10.1371/journal.pgen.1000840)
Supplement: Table S8 — Top candidates identified in both the PAML test on coding sequences and our test for selective sweeps (recent positive selection). (0.05 MB DOC) [file pgen.1000840.s015.doc]

| Ensembl ID | chr | start | end | Human *K* | Pan *K* | Pongo *K* | Macaque *K* | 2ΔL≥100 in lineage |
| --- | --- | --- | --- | --- | --- | --- | --- | --- |
| ENSG00000116560 | 1 | 35421790 | 35431322 | 0 | 0.3176 | 0.2978 | 0 | macaque |
| ENSG00000116819 | 1 | 35811558 | 35833512 | 0 | 0.3798 | 0.0048 | 0.932 | macaque |
| ENSG00000092847 | 1 | 36107996 | 36162486 | 0 | 0.9554 | 0 | 0.5388 | macaque |
| ENSG00000077254 | 1 | 77934264 | 77998125 | 0 | 0.2916 | 0.4824 | 0.1298 | macaque |
| ENSG00000138434 | 2 | 182464840 | 182503707 | 0 | 0.6654 | 0.0038 | 0.3182 | macaque |
| ENSG00000113163 | 5 | 74702684 | 74843719 | 0 | 0.771 | 0.1596 | 0.0354 | pongo, macaque |
| ENSG00000189045 | 5 | 74951884 | 75002621 | 0 | 0.8956 | 0.0256 | 0.6354 | macaque |
| ENSG00000044090 | 6 | 43113336 | 43129632 | 0 | 0 | 0 | 0.4116 | pongo |
| ENSG00000005700 | 6 | 82936675 | 83014190 | 0 | 0.0778 | 0.382 | 0 | macaque |
| ENSG00000185737 | 10 | 83624786 | 84736913 | 0 | 0.3098 | 0.9664 | 0.4132 | pongo |
| ENSG00000075826 | 10 | 102236393 | 102269585 | 0 | 0.363 | 0.7336 | 0 | macaque |
| ENSG00000111731 | 12 | 22492808 | 22588719 | 0 | 0.3192 | 0 | 0.863 | macaque |
| ENSG00000138629 | 15 | 72525371 | 72540576 | 0 | 0.4926 | 0.1838 | 0.4302 | macaque |
| ENSG00000186260 | 16 | 14072697 | 14268130 | 0 | 0.1898 | 0.6442 | 0.1864 | pongo, macaque |
