# Supplementary material for: Human and Non-Human Primate Genomes Share Hotspots of Positive Selection
Source: PLoS Genet. 2010 Feb 5;6(2):e1000840. doi: 10.1371/journal.pgen.1000840 (PMC2816677; doi:10.1371/journal.pgen.1000840)
Supplement: Table S9 — Ensembl genes observed in a selective sweep only in human. Note that all candidates on chromosome 2 except the first correspond to the lactase locus. (0.05 MB DOC) [file pgen.1000840.s016.doc]

| Ensembl ID | HGNC symbol | chr | start | end | Refseq annotation (extract) |
| --- | --- | --- | --- | --- | --- |
| ENSG00000142686 | C1orf216 | 1 | 35952064 | 35957377 | Interacts with Usher syndrome 1C binding protein 1 (USHBP1) and keratin 15, who also mutually interact. |
| ENSG00000116157 | GPX7 | 1 | 52840632 | 52847310 | Plays an essential role in breast cancer cells in alleviating oxidative stress generated from polyunsaturated fatty acid metabolism |
| ENSG00000182183 | FAM159A | 1 | 52871654 | 52907925 | - |
| ENSG00000124356 | STAMBP | 2 | 73909594 | 73954268 | Associates with the SH3 domain of STAM |
| ENSG00000176601 | YSK4 | 2 | 135438743 | 135498718 | - |
| ENSG00000082258 | CCNT2 | 2 | 135392863 | 135431056 | The p-TEFb complex containing this cyclin was reported to interact with, and act as a negative regulator of human immunodeficiency virus type 1 (HIV-1) Tat protein. |
| ENSG00000115839 | RAB3GAP1 | 2 | 135526323 | 135644048 | Implicated in regulated exocytosis of neurotransmitters and hormones. Homozygous mutant in microcephaly syndrome. |
| ENSG00000144224 | UBXN4 | 2 | 136215659 | 136259103 | UBXD2 may be involved in Alzheimer's disease. |
| ENSG00000115866 | DARS | 2 | 136380724 | 136459692 | Aspartyl-tRNA synthetase |
| ENSG00000109686 | SH3D19 | 4 | 152260886 | 152367022 | SH3 domain containing 19 |
| ENSG00000187772 | LIN28B | 6 | 105511616 | 105637899 | Critical role of LIN28B during development and tumorigenesis |
| ENSG00000112276 | BVES | 6 | 105651390 | 105691236 | may play an important role in development of cardiac and skeletal muscle. |
| ENSG00000132429 | POPDC3 | 6 | 105712476 | 105734563 | may play an important role in cardiac and skeletal muscle during development |
| ENSG00000139173 | TMEM117 | 12 | 42516168 | 43069807 | transmembrane protein 117 |
| ENSG00000167014 | C15orf43 | 15 | 43036192 | 43058711 | - |
| ENSG00000167434 | CA4 | 17 | 55582079 | 55591683 | Proteins of this family participate in respiration, calcification, acid-base balance, bone resorption, and the formation of aqueous humor, cerebrospinal fluid, saliva, and gastric acid. It may have a role in inherited renal abnormalities of bicarbonate transport |
| ENSG00000141371 | C17orf64 | 17 | 55854652 | 55863563 |  |
| ENSG00000062725 | APPBP2 | 17 | 55875302 | 55958362 | The beta-amyloid precursor protein is thought to play a role in the pathogenesis of Alzheimer's disease. This gene has been found to be highly expressed in breast cancer |
| ENSG00000166960 | C18orf34 | 18 | 28771365 | 29274683 | - |
