# Supplementary material for: Human and Non-Human Primate Genomes Share Hotspots of Positive Selection
Source: PLoS Genet. 2010 Feb 5;6(2):e1000840. doi: 10.1371/journal.pgen.1000840 (PMC2816677; doi:10.1371/journal.pgen.1000840)
Supplement: Text S1 — Weighing scheme evaluation. (0.02 MB DOC) [file pgen.1000840.s017.doc]

A weighing scheme was introduced to eliminate the effect of biological and technical factors known to affect genetic diversity and/or the ability to identify it. In order to evaluate the efficiency of this weighing scheme, we simulated a number of factors affecting the observed rate of heterozygosity in individual genomes generated by forward population simulations (panmictic population, Text S2). The effect of simulated factors was predefined according to a specific SNP calling probability law. For example, read coverage can be simulated considering that during the whole genome sequencing process both chromosomes of a diploid individual have equal chance of being sampled. The probability *F*(*n*) that both chromosomes were sequenced and hence that heterozygosity is visible at a given position then only depends on the number *n* of reads aligning to this position and is given by:

*F*(n) = 1-2(0.5)^n^

Factors were simulated to obtain different patterns of heterogeneity along 1.1 Gb chromosomes. Regions were defined along chromosomes with sizes following a predefined gamma distribution, and average values taken by the simulated factor within each region were themselves generated according to a second predefined gamma distribution. More or less asymmetric gamma distributions (compared to a symmetric gamma distribution with a very large shape parameter value) could be used to generate different levels of heterogeneity.

The test was performed using 200 kb windows and a 20 fold greater genomic background window sliding every 10 kb (see Methods), first without correcting for simulated factors to obtain their correlations with *K*, and then with correction to verify whether these correlations were removed or not. We could verify that the simple weighing scheme we introduced removes nearly entirely the correlation of *K* with biasing factors in a diverse range of situations (Table S1).
