# Supplementary material for: Human and Non-Human Primate Genomes Share Hotspots of Positive Selection
Source: PLoS Genet. 2010 Feb 5;6(2):e1000840. doi: 10.1371/journal.pgen.1000840 (PMC2816677; doi:10.1371/journal.pgen.1000840)
Supplement: Text S2 — Forward population simulations. (0.03 MB DOC) [file pgen.1000840.s018.doc]

We used the forward population simulator Fregene to evaluate the performance of our test under realistic selection and recombination conditions [37-39]. Two populations with selection were considered, one panmictic and one simulating the major changes in human demography since the emergence of modern human (see references 38 and 39 for additional details on population parameters not given below).

In the first panmictic population, 6 Gb of sequences were simulated with a rescaling factor of 10 in 25,000 individuals during 150,000 generations. Rescaling did not affect the results, as shown by comparisons of the results obtained with 6 Gb with rescaling and 2 Gb without rescaling (data not shown). Randomly occurring advantageous mutations were included, with 4.5 per million new mutations having their selection coefficient s=0.01, and 0.5 per million with s=0.1 and all other mutations with s=0. These values of s represent only strongly or very strongly advantageous mutations with 2*Ne*s being well over 100 (*Ne* is the effective population size). Little power is generally expected in order to identify weakly advantageous mutations (2*Ne*s<100). Dominance coefficients of advantageous mutations were modelled using a normal distribution with mean 0.5 and standard deviation 0.1.

Realistic recombination was modelled following reference 39. At 125,000 generations, patterns of recombination were modified every 25,000 generations to account for the turnover of recombination hotspots over time.

The second population is meant to model human demographic evolution and uses the parameters defined in references 38 and 39. It starts with a constant size of 25,000 African individuals evolving for 125,000 generations, followed by instantaneous expansion to 48,000 individuals and 17,000 further generations. The Out-Of-Africa event is then modelled by an instantaneous bottleneck with 4,080 individuals founding non-African populations. The ancestral non-African population then expands to 15,410 individuals and evolves for 3,500 generations. At this stage, the European and Asian populations split with 320 and 1,041 individuals founding the European and Asian populations, respectively. Both populations expand back to 15,410 individuals with symmetric migration [38] during 2,000 generations and finally 350 generations with no migration. Rescaling was shown to have no effect on final results (data not shown). We ran our test on randomly sampled European individuals at the end of the demographic process. The whole process was repeated as many times as needed to reach a total size of 6 Gb simulated sequences.

We ran our test with 200 kb windows and 20 fold greater genomic background (see Methods), assuming constant divergence and constant read coverage providing complete allelic sampling along chromosomes. Assuming constant divergence is justified by the fact that the power of HKA-based approaches depends mostly on the decrease of heterozygosity due to a selective sweep [40]. Estimates of power and specificity may however be overestimated and underestimated, respectively, when compared to real test conditions. However this is only slightly the case as long as real tested genomes have high coverage and good allele sampling, as in our analysis. In particular, this should not affect the differences in power and specificity between the two population models.

The power and specificity of our test were estimated using 200 kb windows sliding every 10 kb with one, two or 20 randomly sampled individuals. When using several individuals, the statistic *K* (see Methods) is first calculated on each individual genome separately and then averaged over all individuals. This makes the correction for read coverage bias very easy, in particular when individual genomes were not sequenced with the same technique and/or have very different average genome-wide coverage.

The performance of the test was evaluated under both the panmictic and human population models, using one (Fig. S1), two (Fig. S2) or twenty (Fig. S3) individuals and thresholds of *K* ranging from 0 to 0.2. Using one or two individuals yields substantial rates of false positives (a false positive at threshold *K=k* is a region with windows showing a *K* comprised between *k*-0.005 and *k*+0.005, and in which no fixation of advantageous mutation occurred within the last 10,000 generations). Human demography only had a moderate effect on the test. Surprisingly however, the initially lower rate of false positives becomes higher in the panmictic than in the human demography population as power increases. This is due to the fact that selective sweeps in the panmictic population tend to decrease heterozygosity over a wider scale, creating more regions with low *K* that do not overlap the advantageous mutation. Notably, the latter makes our false positives estimates conservative. Power is however very low in regions with high recombination rates, a result that is expected when using physical distances with no correction for recombination (62% power below the first recombination quartile, but only 21% power above the last recombination quartile when using two individuals, *K*≤0.05 and s=0.01). This limitation may be at least partially overcome in the future when tens of individuals become available in diverse species, enabling the estimation of precise recombination rates and the replacement of physical distances with genetic distances in our method.

Power is systematically greater to detect fixed mutations with s=0.1 than those with s=0.01, reflecting an expected detection bias of our method towards more strongly advantageous mutations.

Power is increased and the rate of false positives strongly decreased when using 20 individuals instead of only one or two, showing the potential of the approach as many more high coverage genomes become available. Indeed, a power of 70% to detect advantageous mutations with *s*=0.01 that fixed within the 2,500 last generations corresponds to a false positive rate of 35% in the human-demography population (note that these values do not match what can be observed from Fig. S3 due to the more restrictive definition of false positives used in the latter case). Interestingly, power and specificity are very weakly affected by demographic changes in the human population when using 20 individuals.

Power was systematically very low to detect advantageous mutations in the process of being fixed at frequencies lower than 0.9 (data not shown). Although the two population models we used represent a very small fraction of all possible demographies, the method thus seems relatively robust to demographic changes to detect recently fixed advantageous mutations.
