# Supplementary material for: Human and Non-Human Primate Genomes Share Hotspots of Positive Selection
Source: PLoS Genet. 2010 Feb 5;6(2):e1000840. doi: 10.1371/journal.pgen.1000840 (PMC2816677; doi:10.1371/journal.pgen.1000840)
Supplement: Text S3 — Distinguishing between hotspots and coldspots models. (0.03 MB DOC) [file pgen.1000840.s019.doc]

Two scenarios can explain our observations that there exist more genes in putative sweeps in three or more primates simultaneously than expected by chance. In the first scenario, the excess may be a consequence of the presence of coldspots of positive selection, where a minority of genes are constantly under low rates of positive selection in all the lineages studied, leading to selective sweeps concentrating on the remaining genes. The alternative scenario is that the primate genome contains hotspots of positive selection where a minority of genes has an accelerated rate of positive selection independently in different primate lineages when compared to the rest of the genome. We used a simple analytical model to distinguish between these two possible explanations. Our model considers two classes of genes: (1) a proportion *α* of genes that are in sweeps at frequency *w* and (2) a proportion (1- *α*) of genes that are in sweeps at a higher frequency *λw*, with *λ*>1. When *α*<0.5, the excess of co-occurrences in more than three genomes can be mainly attributed to selective sweeps steering clear of a small proportion of coldspots, and thus mainly occurring in the rest of the genome (“coldspot” scenario). In contrast when *α*>0.5, the excess of co-occurrences can be mainly attributed to selective sweeps concentrating on a small number of hotspots (“hotspot” scenario). The number of genes observed in sweeps *n_e_* (*K*≤0.05) in human and at least two other primates is therefore the sum of three components: (1) *n_cold_*, the number of genes seen in three or more genome and belonging to proportion *α* of genes, (2) *n_hot_*, the number of genes that are seen in sweeps in three or more genomes due to the proportion 1-*α* of genes and (3) *n_false_* the number of false positives that are contributed by any gene in the genome. *N* is the total number of four-way orthologous genes tested. The values *p_1_* (0.177), *p_2_* (0.210), *p_3_* (0.237) and *p_4_* (0.041) are the proportions of genes with *K*≤0.05 respectively in chimpanzee, orangutan, macaque and human. The *f_n_* and *f_h_* parameter are the rates of false positives in non-human primates and in human, respectively (rates of false positives are considered separately in non-human primates and in human due to the difference between *p_1_*, *p_2_*, *p_3_* on the one hand and *p_4_* on the other hand).

Then the expected number of genes significant in human and at least two other primates *n_e_* is given by:

***n_e_ = n_cold +_ n_hot +_ n_false_***

with

***n_cold_= Nα* ( *p_l1_p_l2_p_l4_ + p_l1_p_l3_p_l4_ + p_l2_p_l3_p_l4_*)**

***n_hot_ = N*(*1-α*) *λ^3^*( *p_l1_p_l2_p_l4_ + p_l1_p_l3_p_l4_ + p_l2_p_l3_p_l4_*)**

***n_false_ = N*( *p_f1_p_f2_p_f4_ + p_f1_p_f3_p_f4_ + p_f2_p_f3_p_f4_*)**

and

***p_f1_ = p_1_f_n_ , p_f 2_= p_2_f_n_ , p_f 3_= p_3_f_n_ , p_f4_ = p_4_f_h_***

***p_l1_ =* (*p_1_-p_f1_*)/(*α + λ* (1 *- α*))**

***p_l2_ =* (*p_2_-p_f2_*)/(*α + λ* (1 *- α*))**

***p_l3_ =* (*p_3_-p_f3_*)/(*α + λ* (1 *- α*))**

***p_l4_ =* (*p_4_-p_f4_*)/(*α + λ* (1 *- α*))**

The proportions *p_l1_*, *p_l2_*, *p_l3_* and *p_l4_* are the proportions of significant genes that are in sweeps at a frequency *w* respectively in chimpanzee, orang-utan, macaque and human. The proportions *p_f1_*, *p_f2_*, *p_f3_* and *p_f4_* are the proportions of significant genes that are false positives respectively in chimpanzee, orangutan, macaque and human.

The average random number of co-occurring genes *n_r_* is given by:

***n_r_ = N*(*p_1_p_2_p_4_ + p_1_p_3_p_4_ + p_2_p_3_p_4_*)**

The relative excess *E* when compared to random is then given by:

***E =* (*n_e_ – n_r_*)/*n_r_***

We explored the different combinations of *α, λ* (ranging from 1 to 1,000 with steps of 0.1 from 1 to 10, steps of 1 from 10 to 20, steps of 5 from 20 to 100, and steps of 50 from 100 to 1000)*, f_n_* and *f_h_* for which *E* ranges from the minimal value that is still significant at the 5% level (*E*=0.22) to the observed value (*E*=0.34). Figure S6 shows the variation of the theoretical fraction of false positives that are obtained when *α* varies from 0 to 1, as long as *E* remains within the [0.22-0.34] range. In a coldspot model, a minor proportion of genes in the genome evolves with rare positive selection. Figure S6 shows that if *α*<0.5, then *f_n_*<0.4. Because *p_1_*, *p_2_* and *p_3_* are measured in individual genomes, we expect the proportion of false positive to be relatively high, in the range from 50% to 80% or even more (Text S2). A rate of false positives *f_n_*<0.4 is thus much lower than the range that can reasonably be expected. The high level of co-occurrence between genes in candidate sweeps that we observe is thus best explained by the hotspots model, where a minority of genes has an accelerated rate of positive selection in all the primate genomes tested here.
